# Palladium supported magnetic Fucus Vesiculosus extract as a natural and novel catalyst for the synthesis of *N*-alkyl-2-(4-methyl-1-oxoisoquinolin-2(1*H*)-yl)-2-phenylacetamide derivatives

**DOI:** 10.1038/s41598-023-28121-1

**Published:** 2023-01-23

**Authors:** Faeze Yousefnejad, Saeed Bahadorikhalili, Maryam Esmkhani, Mehdi Adib, Shahrzad Javanshir, Samanehsadat Hosseini, Bagher Larijani, Mohammad Mahdavi

**Affiliations:** 1 School of Chemistry, College of Sciences, University of Tehran, Tehran, Iran; 2 Department of Electronic Engineering, Universitat Rovira i Virgili, 43007 Tarragona, Spain; 3 Department of Chemistry, Iran University of Science and Technology, Tehran, Iran; 4 Shahid Beheshti University of Medical Sciences, Tehran, Iran; 5 Endocrinology and Metabolism Research Center, Endocrinology and Metabolism Clinical Sciences Institute, Tehran University of Medical Sciences, Tehran, Iran

**Keywords:** Chemistry, Nanoscience and technology

## Abstract

In this paper, a novel catalyst is introduced based on the immobilization of palladium onto magnetic Fucus Vesiculosus extract (Pd@m*FuVe* catalyst). For the synthesis of Pd@m*FuVe* catalyst, Fucus Vesiculosus extract is obtained from the plant source, followed by the synthesis of superparamagnetic iron oxide nanoparticles (SPION) onto the extract. The catalyst is characterized by several methods, including scanning electron microscopy (SEM), energy-dispersive X-ray spectroscopy (EDS), FT-IR spectroscopy, vibrating sample magnetometer (VSM), powder X-ray diffraction analysis (XRD), and inductively coupled plasma (ICP). The activity of Pd@m*FuVe* catalyst is studied in the synthesis of *N*-alkyl-2-(4-methyl-1-oxoisoquinolin-2(1*H*)-yl)-2-phenylacetamides. The products were synthesized in three steps, the synthesis of 2-iodobenzoic acid from 2-aminobenzoic acid, which participated in a multicomponent reaction with allylamine, aldehydes, and isocyanides, followed by a cyclization reaction, catalyzed by Pd@m*FuVe* catalyst. The product yields are high and the catalyst showed good reusability after 5 sequential runs. The most significant, Pd@m*FuVe* catalyst is fabricated from a plant extract source as a green support for the catalyst.

## Introduction

Catalytic processes have attracted interest, due to their advantageous benefits, including higher yields of the products, lower reaction temperatures, milder reaction conditions, and shorter reaction times. Regarding the advantages of the catalysts, their application has been increasingly grown in various industries^1–5^. However, many of the catalysts, which are used in chemical transformations still suffer a number of drawbacks such as hard isolation from the reaction mixture, fabrication from toxic materials, low reusability, and the need for harsh reaction conditions^6–8^. Therefore, several efforts have been focused on the introduction of novel catalysts to reduce the disadvantages of previous catalysts. in this way, an interesting approach is the fabrication of catalysts from natural sources^9–12^. In this way, several novel solid supports have been used for the immobilization of the catalyst. Silica^13^, H_4_[SiW_12_O_40_]^14^, Graphene Oxide^15,16^, and SiO_2_–PPA Nanoparticle^17^ are a number of nanoparticles that are used for the fabrication of novel catalysts.

Palladium is a significant transition metal that is extensively used as a catalyst in several chemical transformations. This metal has been used alkoxycarbonylations^18^, reduction of nitroarenes^19^, hydrogenation^20,21^, oxidation^22,23^, and carbon–carbon bond formation^24–27^. In addition, this metal has been used in non‐fullerene‐based organic solar cells^28^, and fuel cells^29^. For increasing the efficiency and reusability of the palladium catalyst, an efficient way is to support it onto solid materials. However, the use of palladium in heterogeneous form causes some disadvantages, especially the need to use a higher temperature and harsher reaction conditions. To overcome this issue, an efficient approach is to use nanomaterials as support for palladium catalysts. By this approach, the catalyst benefits from the high efficiency of homogeneous catalysts and the ease of isolation of heterogeneous ones. Various nanoparticles have been used as support for palladium. Gold^30^, porous polymers^31^, magnetic graphene oxide^32^, magnetic iron oxide^33^, and silica nanoparticles^34^ are a number of nanomaterials that are used as support for palladium in different chemical reactions.

**Scheme 1:**
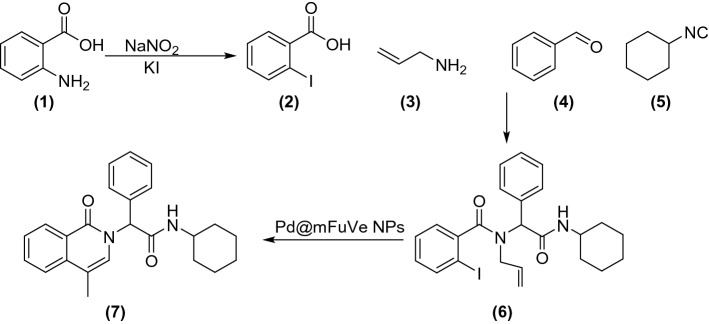
Synthesis steps of the synthesis of *N*-alkyl-2-(4-methyl-1-oxoisoquinolin-2(1*H*)-yl)-2-phenylacetamide derivatives.

Multicomponent reactions are of high interest, due to several advantages including fast reaction performance, high yield of the products, and high atom efficiency^35–38^. Therefore, these kinds of reactions have been successfully applied for the synthesis of various compounds with complex structures^39,40^. Regarding the advantages of multicomponent reactions and the high efficiency and benefits of supported catalysts, we report a novel catalyst based on the immobilization of palladium onto Fucus Vesiculosus extract for the synthesis of *N*-alkyl-2-(4-methyl-1-oxoisoquinolin-2(1*H*)-yl)-2-phenylacetamide derivatives. The synthesis is based on a three-step reaction via Ugi multicomponent reaction. Fucus Vesiculosus extract supported catalyst is used for the cyclization reaction between alkene bond and aromatic carbon iodide. The advantage of this report is the design, synthesis, characterization, and application of the recyclable catalyst based on a plant source. The synthesis steps are presented in Scheme 1. Based on the reactions, in the first step 2-aminobenzoic acid (**1**) is converted to 2-iodobenzoic acid (**2**) using sodium nitrite and potassium iodide. Ugi reaction of 2-iodobenzoic acid, allyl amine (**3**) benzaldehydes (**4**), and isocyanides (**5**) lead to the formation of *N*-allyl-*N*-(2-(alkylamino)-2-oxo-1-phenylethyl)-2-iodobenzamide (**6**). Catalytic cyclization of compound **6** in the presence of Pd@m*FuVe* NPs as the catalyst leads to the synthesis of the desired *N*-alkyl-2-(4-methyl-1-oxoisoquinolin-2(1*H*)-yl)-2-phenylacetamide derivatives (**7**).

## Results and discussions

**Scheme 2:**
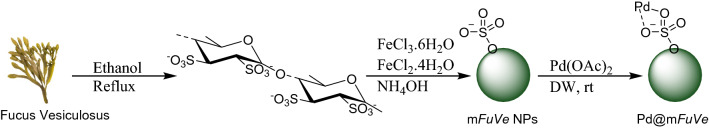
The preparation steps of Pd@m*FuVe* NPs catalyst.

In this paper, Pd@m*FuVe* NPs catalyst is fabricated based on the immobilization of palladium onto magnetic extract as a natural support for the catalyst. For this purpose, Fucus Vesiculosus was ground and the fucoidan was extracted by reflux in ethanol. Magnetic particles were synthesized on the extract by the reaction of Fe^2+^ and Fe^3+^ in a basic medium (m*FuVe* NPs). The palladium catalyst was introduced by the addition of palladium acetate to the aqueous suspension of m*FuVe* NPs. The synthesis steps of Pd@m*FuVe* NPs catalyst are presented in Scheme 2.

**Figure 1:**
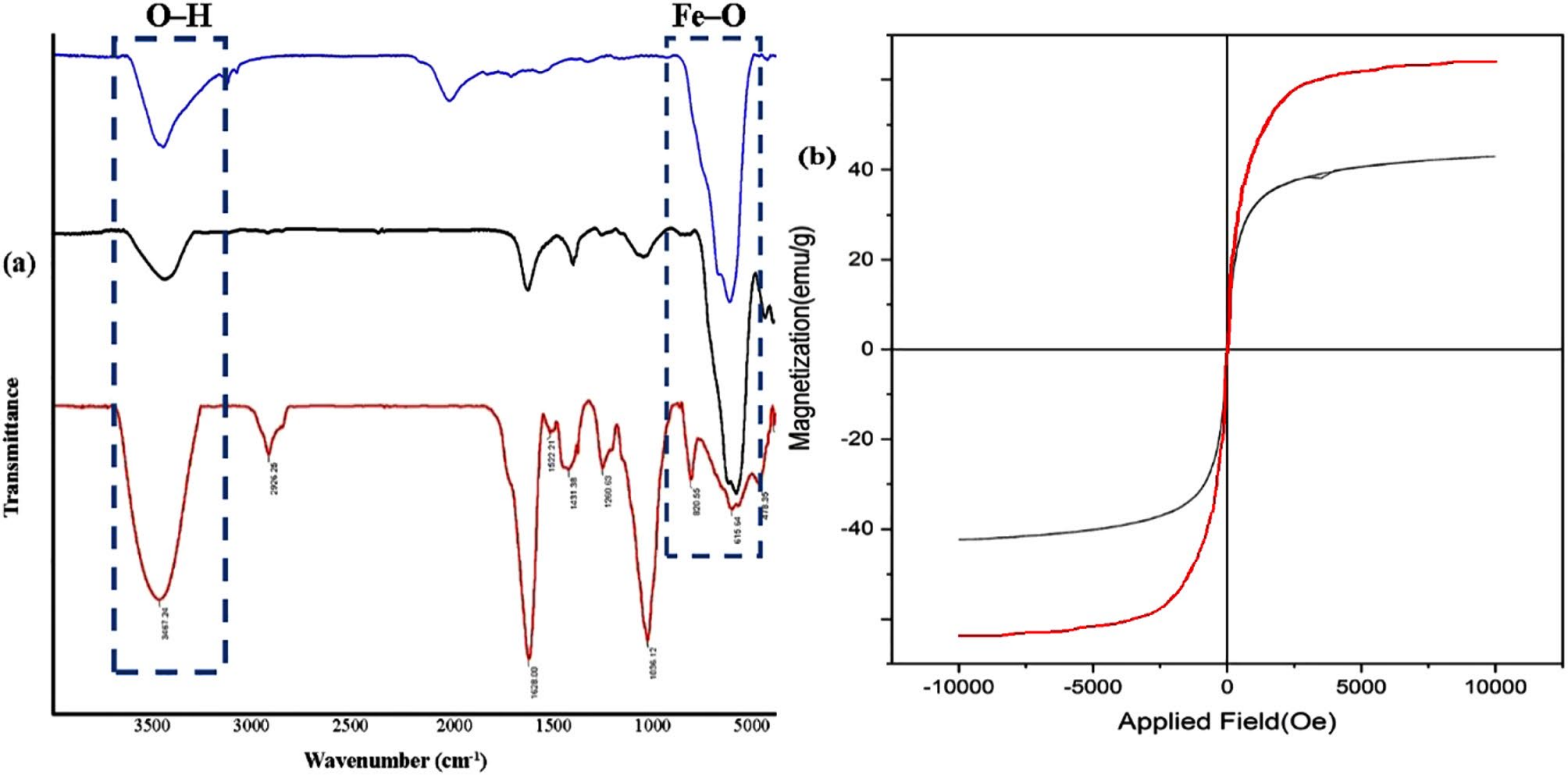
(**a**) FT-IR spectrum of Fe_3_O_4_ (blue) m*FuVe* NPs (black) Pd@m*FuVe* NPs catalyst (red); and (**b**) VSM graph of Fe_3_O_4_ (red) Pd@m*FuVe* NPs catalyst (black).

The synthesis of the catalyst was characterized by several characterization techniques. Based on the characteristic peaks in FT-IR spectra of m*FuVe* NPs Pd@m*FuVe* NPs catalyst in Fig. 1a, based on the FT-IR spectra, Pd@m*FuVe* NPs catalyst has successfully been synthesized. The bonds at 3467 and 2926 cm^−1^ correspond O–H and aliphatic C–H vibrations, respectively. In addition, the peak at 575 cm^−1^ could be correlated to the Fe–O vibrations. The presence of magnetic iron oxide with superparamagnetic behavior is approved by VSM method (Fig. 1b).

**Figure 2:**
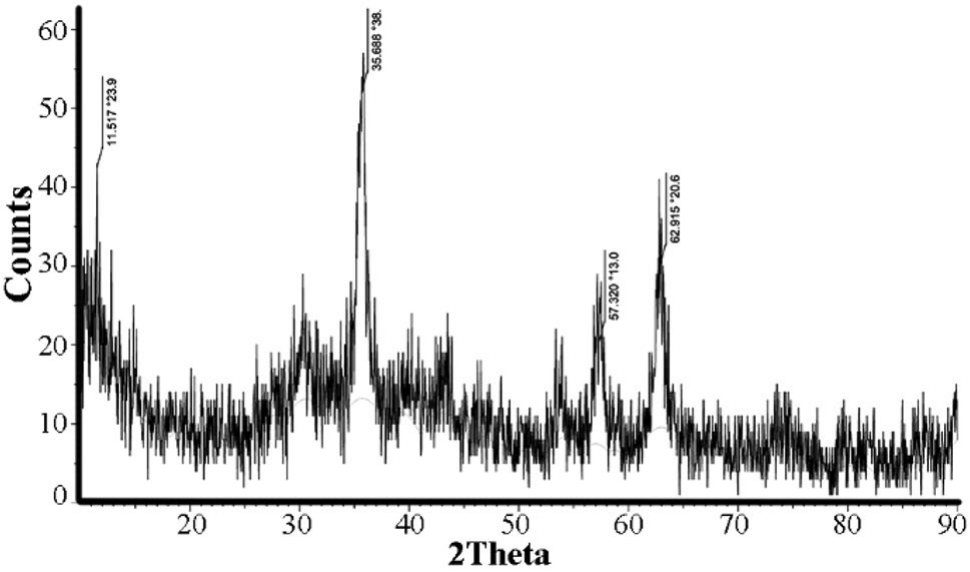
XRD pattern of Pd@m*FuVe* NPs catalyst.

The crystalline structure of Pd@m*FuVe* NPs catalyst was studied by XRD method. The diffraction pattern of Pd@m*FuVe* NPs catalyst is presented in Fig. 2. The diffraction patterns at 2θ = 36, 57, and 63° are in agreement with the desired structure of the catalyst.

**Figure 3:**
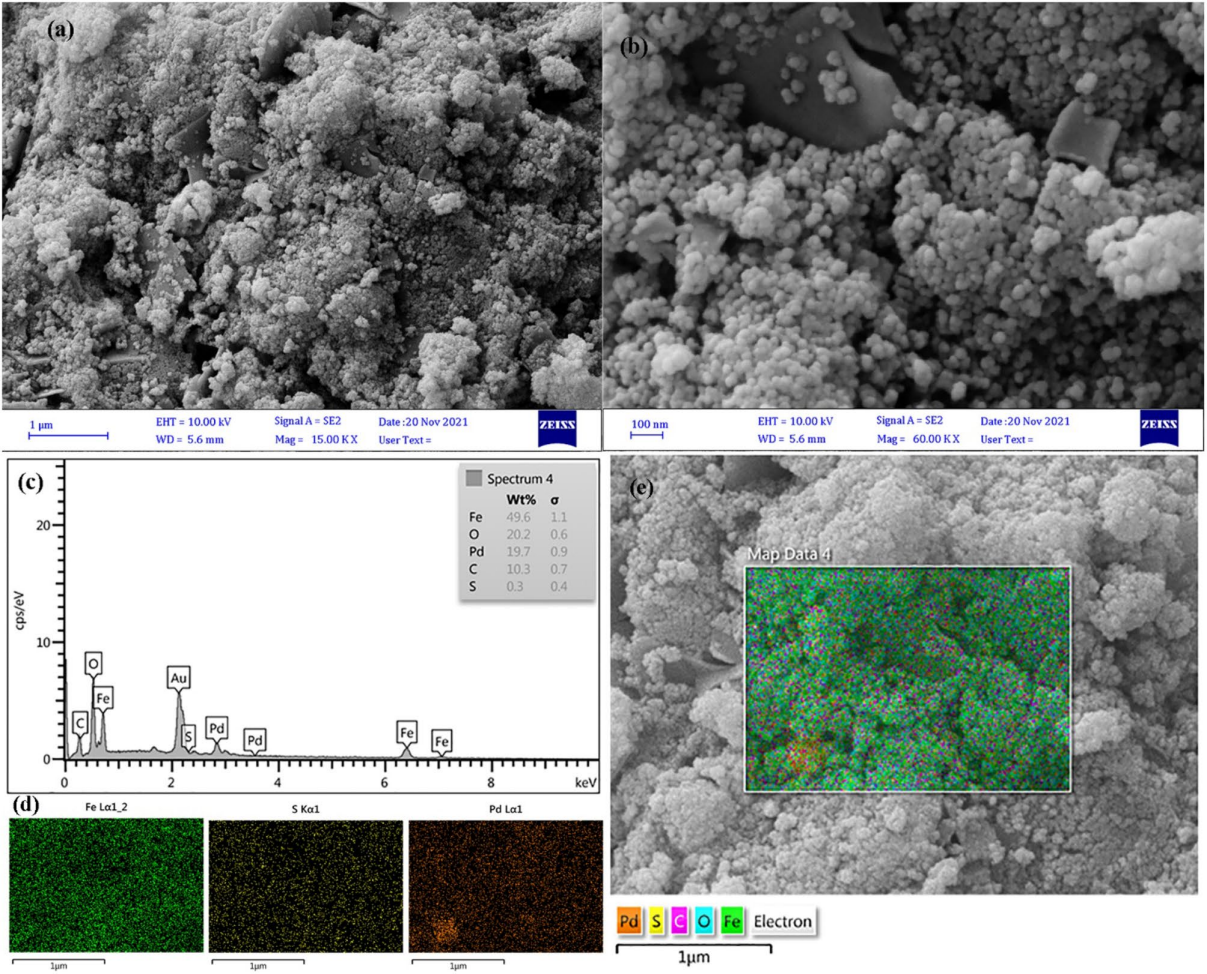
(**a,b**) SEM images of Pd@m*FuVe* NPs catalyst in different magnifications; (**c**) EXS; and (**d,e**) SEM mapping of Pd@m*FuVe* NPs catalyst.

The microstructure of Pd@m*FuVe* NPs catalyst was studied by SEM microscopy. The formation of magnetic iron oxide nanoparticles on the Fucus Vesiculosus extract could be observed. The nanoparticles have spherical morphology with a diameter of 50–60 nm. The morphology of the nanoparticles is uniform with narrow particle size distribution. SEM images with different magnifications are presented in Fig. 3a,b. The presence of Fe, C, O, and S elements was observed in EDS results, which confirm the structure of the catalyst. In addition, the presence of palladium could be confirmed by this method (Fig. 3c). For the measurement of the Pd content in the structure of Pd@m*FuVe* NPs catalyst, the ICP method was used. The Palladium content was measured to be 21.372 mmol/g of the catalyst. The distribution of the catalyst was studied by SEM mapping. The uniform distribution of iron oxide and palladium on the surface of Pd@m*FuVe* NPs catalyst could be seen in Fig. 3d,e.

Based on the characterization results of Pd@m*FuVe* NPs catalyst, the catalytic activity of this catalyst was evaluated in the synthesis of *N*-alkyl-2-(4-methyl-1-oxoisoquinolin-2(1*H*)-yl)-2-phenylacetamide derivatives. The products are obtained in three steps. In the first step, 2-aminobenzoic acid (**1**) reacts with sodium nitrite and potassium iodide to form 2-iodobenzoic acid (**2**), which undergoes Ugi multicomponent reaction with allylamine (**3**), an aldehyde (**4**) and an isocyanide (**5**). *N*-allyl-*N*-(2-(alkylamino)-2-oxo-1-phenylethyl)-2-iodobenzamide (**6**), the product of the Ugi multicomponent undergoes a cyclization reaction in the presence of Pd@m*FuVe* NPs catalyst. For finding the optimal reaction conditions, the cyclization reaction of *N*-allyl-*N*-(2-(cyclohexylamino)-2-oxo-1-phenylethyl)-2-iodobenzamide was selected as a model reaction and the yield of the product **7** was regarded for the selection of the best reaction conditions. For this purpose, the reaction was performed in different solvents in the presence of different amounts of catalyst. The results are presented in Table 1. It could be observed that the presence of Pd@m*FuVe* NPs catalyst is essential for the reaction performance. Product **7** has not formed in the presence of m*FuVe* NPs, which shows that the existence of palladium is essential for the reaction. The turnover number (TON) and turnover frequency (TOF) of the catalyst was calculated for the optimized conditions. The TON and TOF were measured as 1660 and 553, respectively.

**Table 1:** Optimization conditions of the synthesis of *N*-cyclohexyl-2-(4-methyl-1-oxoisoquinolin-2(1*H*)-yl)-2-phenylacetamide.

Entry	Solvent	Base	Amount of catalyst (mol %)	Isolated yield (%)
1	H_2_O	K_2_CO_3_	5	12
2	EtOH	K_2_CO_3_	5	30
3	DMF	K_2_CO_3_	5	83
4	DMSO	K_2_CO_3_	5	75
5	DMF	Et_3_N	5	40
6	DMF	Pyridine	5	32
7	DMF	NaOH	5	58
8	DMF	K_2_CO_3_	0	0
9	DMF	K_2_CO_3_	3	63
10	DMF	K_2_CO_3_	8	83
11	DMF	K_2_CO_3_	m*FuVe* NPs (100 mg)	0

Reaction conditions: Compound **6** (0.3 mmol), base (0.6 mmol), tetrabutylammonium bromide (0.9 mmol), solvent (1 mL) Pd@m*FuVe* catalyst, 75 °C.

Based on the optimal reaction conditions, the scope of the reaction was studied by using different isocyanides and aldehydes. In all cases, the products were formed in very good isolated yields. The structure of the products and their yields are presented in Table 2.

**Table 2:** Optimization conditions of the synthesis of *N*-cyclohexyl-2-(4-methyl-1-oxoisoquinolin-2(1*H*)-yl)-2-phenylacetamide.

Entry	Compound	Structure	Isolated yield (%)
1	**7a**		83
2	**7b**		77
3	**7c**		81
4	**7d**		80
5	**7e**		75
6	**7f**		79
7	**7g**		76
8	**7h**		82
9	**7i**		85
10	**7j**		76

Reaction conditions: Compound **6** (0.3 mmol), potassium carbonate (0.6 mmol), tetrabutylammonium bromide (0.9 mmol), DMF (1 mL) Pd@m*FuVe* catalyst, 75 °C.

**Figure 4:**
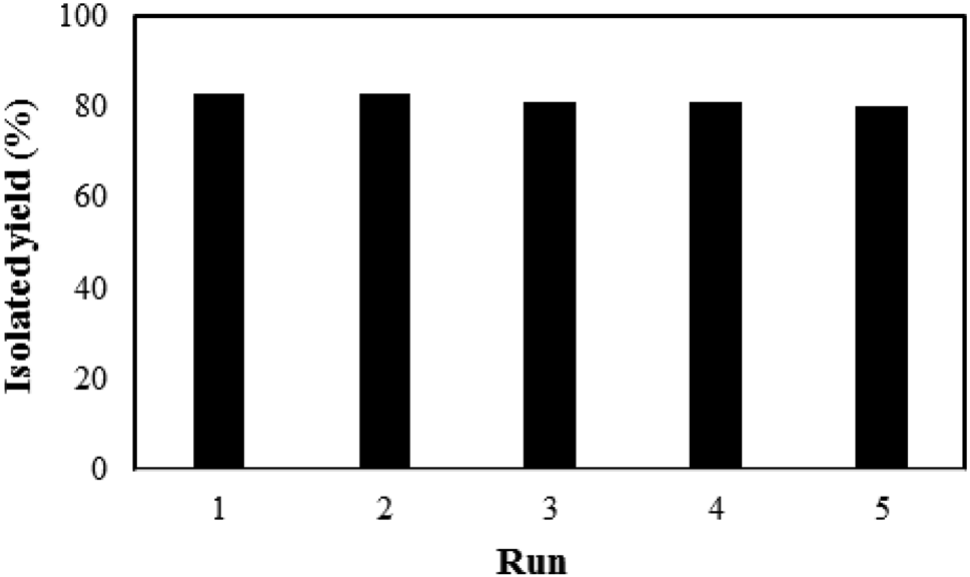
The recovery results of Pd@m*FuVe* catalyst.

As an advantage, the Pd@m*FuVe* catalyst showed very good reusability. To study the reusability of the catalyst, Pd@m*FuVe* catalyst was isolated from the reaction mixture using an external magnet and was used in the next reaction under optimized conditions. This protocol was repeated for 5 sequential reactions for the synthesis of compound **7a**. The results of the recovery of the catalyst are presented in Fig. 4 and confirm the reusability of Pd@m*FuVe* catalyst. The hot filtration test was performed to better study the role of the catalyst in the synthesis of compounds **7a**. for this purpose, the catalyst was separated from the reaction mixture before the completion of the reaction was stirred under optimized conditions for more 4 h and no increase was observed in the conversion of compound **6a** to **7a**. In addition, after the separation of the catalyst from the reaction mixture, the solution was studied by ICP and the results confirmed no palladium leaching from the catalyst.

## Experimental

### General remarks

All the chemicals and reagents were purchased from Merck (Germany) and Sigma (USA) and used as received without any further purifications. The melting point of the products was measured by an electrothermal 9100 instrument. ^1^H NMR and ^13^C NMR spectra were recorded on a Bruker FT-400 in 400 and 101 MHz for ^1^H NMR and ^13^C NMR, respectively using TMS as internal standard. A Shimadzu FT-IR 550 spectrometer was used for recording FT-IR spectra. The samples were prepared as disks using KBr. An Agilent Technology (HP) mass spectrometer was used for recording mass spectra. The instrument was operated at an ionization potential of 70 eV. For the elemental analysis of the samples, an elementar analysen system GmbH VarioEL CHNS mode was utilized. The magnetic properties of the samples were measured using VSM, model BHV-55, Riken, Japan with a magnetic field up to 10 kOe. A MIRA3 by TESCAN was used to record SEM images. For VSM measurements, a FORCE^+^ VMS was used.

### Synthesis of palladium supported magnetic fucoidan

Fucus Vesiculosus (1.25 g) was weighted and powdered with a ball mill for 5 min in 5 Hz, followed by refluxing in ethanol (96%, 50 mL) for 12 h to give fucoidan as the product. The obtained fucoidan was filtered and dried at 50 °C in the vacuum. Then, fucoidan (0.4 g) was added to deionized water (200 mL) and FeCl_2_·4H_2_O (4 g) and FeCl_3_·6H_2_O (11 g) were added. The mixture was vigorously stirred under an argon atmosphere for 15 h at room temperature to obtain a uniform mixture and then, the temperature was raised to 80 °C. Ammonium hydroxide was added dropwise to the reaction mixture for 1 h until the pH of the mixture stabilized at 12. The reaction mixture was stirred for more than 45 min under argon flow at 80 °C. The mixture was cooled to room temperature and the magnetic fucoidan was separated using an external magnet, washed with water and ethanol several times, and dried at 60 °C in a vacuum oven.

Magnetic fucoidan (1 g) was weighted and added to a solution of palladium acetate (0.5 g, 2.25 mmol) in deionized water (30 mL). The mixture was stirred at room temperature for 10 h and Pd@m*FuVe* catalyst was separated using an external magnet. Pd@m*FuVe* catalyst was washed with water and ethanol to remove any possible impurity or physically adsorbed palladium and fried at 60 °C in a vacuum oven for 2 h.

### Synthesis of 2-iodobenzoic acid

A solution of sodium nitrate (12 mmol, 1.02 g) in water (4 mL) was added dropwise to a solution of anthranilic acid (10 mmol, 1.37 g) in concentrated hydrochloric acid (10 eq) at 0 °C for 30 min. the reaction mixture was stirred at 0 °C for more 30 min and then, a pre-cooled solution of potassium iodide (15 mmol, 2.49 g) in water (4 mL) was added dropwise during 20 min. the reaction mixture was stirred for more 1 h at 0 °C. After that, the reaction mixture was heated at 40 °C for 10 min, followed by heating at 80 °C for 10 min. The mixture was cooled to 0 °C and sodium thiosulfate was added to neutralize unreacted iodine. The product was separated from the reaction mixture by filtration and washed with cold water and dried at room temperature overnight.

#### Synthesis of *N*-allyl-*N*-(2-(alkylamino)-2-oxo-1-phenylethyl)-2-iodobenzamide derivatives (6)

Allylamine (2 mmol, 114 mg), 2-iodobenzoic acid (2 mmol, 496 mg), benzaldehyde (2 mmol, 212 mg), and alkyl isocyanide (2 mmol) were added to methanol (2 mmol) and stirred at room temperature for 24 h. the reaction progress was followed by TLC monitoring and after the reaction completion, the reaction was added to water, the precipitate was collected and recrystallized form ethanol.

#### Synthesis of *N*-alkyl-2-(4-methyl-1-oxoisoquinolin-2(1*H*)-yl)-2-phenylacetamide derivatives (7)

Compound **6** (0.3 mmol), potassium carbonate (0.6 mmol, 83 mg), and tetrabutylammonium bromide (0.9 mmol, 290 mg) were added to DMF (1 mL) with Pd@m*FuVe* catalyst (5 mol%). The reaction mixture was stirred at 75 °C and the progress of the reaction was followed by TLC. After the reaction completion, water was added to the reaction mixture and the precipitate was filtered from the reaction mixture. The products were purified by column chromatography on silica using a mixture of petroleum ether/ethyl acetate (9:1, v/v).

### Spectral data of the products

#### *N*-Cyclohexyl-2-(4-methyl-1-oxoisoquinolin-2(1*H*)-yl)-2-phenylacetamide (7a)

White solid; mp 198–202 °C; ^1^H NMR (400 MHz, DMSO-*d*_6_) δ 8.55 (d, *J* = 7.6 Hz, 1H), 8.29 (dd, *J* = 8.1, 1.4 Hz, 1H), 7.77 (ddd, *J* = 8.3, 7.1, 1.4 Hz, 1H), 7.62 (d, *J* = 8.0 Hz, 1H), 7.54 (ddd, *J* = 8.2, 7.1, 1.2 Hz, 1H), 7.39 (ddd, *J* = 13.8, 7.9, 6.2 Hz, 3H), 7.30–7.21 (m, 2H), 6.87 (d, *J* = 1.3 Hz, 1H), 6.80 (s, 1H), 3.66–3.54 (m, 1H), 2.09 (d, *J* = 1.1 Hz, 3H), 1.82–1.48 (m, 5H), 1.25–1.06 (m, 5H). ^13^C NMR (101 MHz, DMSO-*d*_6_) δ 167.22, 161.35, 137.18, 137.12, 133.10, 129.50, 129.07, 128.82, 128.77, 128.22, 127.09, 125.19, 123.81, 110.20, 59.76, 48.52, 32.66, 32.55, 25.60, 24.96, 24.88, 15.69; Anal for C_24_H_26_N_2_O_2_, Calcd: C, 76.98; H, 7.00; N, 7.48; O, 8.54; found: C, 77.01; H, 7.02; N, 7.44; O, 8.53; MS (70 eV): m/z = 374 (M^+^).

#### *N*-Cyclohexyl-2-(4-methoxyphenyl)-2-(4-methyl-1-oxoisoquinolin-2(1*H*)-yl)acetamide (7b)

White solid; mp 218–220 °C; ^1^H NMR (400 MHz, DMSO-*d*_6_) δ 8.49 (d, *J* = 7.7 Hz, 1H), 8.31 (dd, *J* = 8.0, 1.4 Hz, 1H), 7.78 (ddd, *J* = 8.4, 7.1, 1.4 Hz, 1H), 7.63 (d, *J* = 7.9 Hz, 1H), 7.55 (ddd, *J* = 8.1, 7.1, 1.2 Hz, 1H), 7.28–7.18 (m, 2H), 7.03–6.95 (m, 2H), 6.88 (d, *J* = 1.3 Hz, 1H), 6.74 (s, 1H), 3.77 (s, 3H), 3.61 (dtd, *J* = 10.9, 7.0, 3.3 Hz, 1H), 2.11 (d, *J* = 1.2 Hz, 3H), 1.85–1.49 (m, 5H), 1.30–1.10 (m, 5H). ^13^C NMR (101 MHz, DMSO-*d*_6_) δ 167.54, 161.33, 159.60, 137.17, 133.01, 130.58, 128.71, 128.17, 126.99, 125.24, 123.75, 114.85, 110.02, 59.48, 55.61, 48.48, 32.69, 32.56, 25.61, 24.98, 24.89, 15.70; Anal for C_25_H_28_N_2_O_3_, Calcd: C, 74.23; H, 6.98; N, 6.93; O, 11.87; found: C, 74.23; H, 6.98; N, 6.93; O, 11.87; MS (70 eV): m/z = 404 (M^+^).

#### *N*-Cyclohexyl-2-(2-fluorophenyl)-2-(4-methyl-1-oxoisoquinolin-2(1*H*)-yl)acetamide (7c)

White solid; mp 230–232 °C; ^1^H NMR (400 MHz, DMSO-*d*_6_) δ 8.58 (d, *J* = 7.7 Hz, 1H), 8.31 (dd, *J* = 8.1, 1.4 Hz, 1H), 7.80 (ddd, *J* = 8.3, 7.1, 1.4 Hz, 1H), 7.65 (d, *J* = 8.1 Hz, 1H), 7.61–7.54 (m, 1H), 7.49 (tdd, *J* = 7.5, 5.3, 1.9 Hz, 1H), 7.44–7.20 (m, 3H), 6.95 (s, 1H), 6.77 (d, *J* = 1.4 Hz, 1H), 3.63 (dtd, *J* = 10.5, 7.0, 3.8 Hz, 1H), 2.16–2.07 (m, 3H), 1.86–1.51 (m, 5H), 1.32–1.05 (m, 5H). ^13^C NMR (101 MHz, DMSO-*d*_6_) δ 166.63, 160.96, 159.65, 137.19, 133.14, 131.57, 130.61, 128.15, 128.03, 127.19, 125.43, 125.19, 124.24, 124.09, 123.87, 116.26, 110.55, 54.89, 48.57, 32.56, 32.48, 25.58, 24.89, 24.80, 15.65; Anal for C_24_H_25_FN_2_O_2_, Calcd: C, 73.45; H, 6.42; F, 4.84; N, 7.14; O, 8.15; found: C, 73.45; H, 6.42; F, 4.84; N, 7.14; O, 8.15; MS (70 eV): m/z = 392 (M^+^).

#### *N*-Cyclohexyl-2-(4-methyl-1-oxoisoquinolin-2(1*H*)-yl)-2-(*p*-tolyl)acetamide (7d)

White solid; mp 218–220 °C; ^1^H NMR (400 MHz, DMSO-*d*_6_) δ 8.52 (d, *J* = 7.7 Hz, 1H), 8.31 (dd, *J* = 8.1, 1.4 Hz, 1H), 7.79 (ddd, *J* = 8.3, 7.1, 1.4 Hz, 1H), 7.64 (d, *J* = 8.0 Hz, 1H), 7.56 (ddd, *J* = 8.2, 7.1, 1.2 Hz, 1H), 7.29–7.14 (m, 4H), 6.87 (d, *J* = 1.3 Hz, 1H), 6.77 (s, 1H), 3.63 (dtd, *J* = 10.5, 7.0, 3.8 Hz, 1H), 2.32 (s, 3H), 2.11 (d, *J* = 1.1 Hz, 3H), 1.85–1.61 (m, 5H), 1.33–1.01 (m, 5H). ^13^C NMR (101 MHz, DMSO-*d*_6_) δ 170.83, 167.38, 161.33, 138.19, 137.17, 134.04, 133.06, 130.05, 129.08, 128.79, 128.19, 127.04, 125.20, 123.78, 110.07, 60.24, 48.48, 32.66, 32.56, 25.61, 24.96, 24.88, 21.18, 14.56; Anal for C_25_H_28_N_2_O_2_, Calcd: C, 77.29; H, 7.26; N, 7.21; O, 8.24; found: C, 77.29; H, 7.26; N, 7.21; O, 8.24; MS (70 eV): m/z = 388 (M^+^).

#### 2-(3-Bromophenyl)-*N*-cyclohexyl-2-(4-methyl-1-oxoisoquinolin-2(1*H*)-yl)acetamide (7e)

White solid; mp 218–220 °C; ^1^H NMR (400 MHz, DMSO-*d*_6_) δ 8.60 (d, *J* = 7.7 Hz, 1H), 8.31 (dd, *J* = 8.1, 1.4 Hz, 1H), 7.80 (ddd, *J* = 8.3, 7.1, 1.4 Hz, 1H), 7.66 (d, *J* = 8.0 Hz, 1H), 7.63–7.52 (m, 2H), 7.48–7.36 (m, 2H), 7.33–7.25 (m, 1H), 6.95 (d, *J* = 1.3 Hz, 1H), 6.80 (s, 1H), 3.69–3.56 (m, 1H), 2.15 (d, *J* = 1.2 Hz, 3H), 1.87–1.48 (m, 5H), 1.33–1.07 (m, 5H). ^13^C NMR (101 MHz, DMSO-*d*_6_) δ 166.68, 161.32, 139.73, 137.21, 133.20, 131.75, 131.70, 131.68, 128.47, 128.23, 128.09, 127.20, 125.19, 123.87, 122.49, 110.69, 59.24, 48.54, 32.57, 32.47, 25.58, 24.88, 24.82, 15.62; Anal for C_24_H_25_BrN_2_O_2_, Calcd: C, 63.58; H, 5.56; Br, 17.62; N, 6.18; O, 7.06; found: C, 63.62; H, 5.55; N, 6.20; O, 7.01; MS (70 eV): m/z = 452 (M^+^).

#### *N*-Cyclohexyl-2-(4-methyl-1-oxoisoquinolin-2(1*H*)-yl)-2-(3-nitrophenyl)acetamide (7f)

Yellow solid; mp 228–232 °C; ^1^H NMR (400 MHz, DMSO-*d*_6_) δ 8.66 (d, *J* = 7.7 Hz, 1H), 8.33 (dd, *J* = 8.1, 1.4 Hz, 1H), 8.25 (dt, *J* = 6.8, 2.3 Hz, 1H), 8.13 (d, *J* = 2.3 Hz, 1H), 7.85–7.62 (m, 4H), 7.62–7.52 (m, 1H), 7.02 (d, *J* = 1.3 Hz, 1H), 6.92 (s, 1H), 3.67 (tdt, *J* = 11.0, 7.6, 3.8 Hz, 1H), 2.20–2.12 (m, 3H), 1.90–1.54 (m, 5H), 1.35–1.03 (m, 5H). ^13^C NMR (101 MHz, DMSO-*d*_6_) δ 166.44, 161.38, 148.42, 139.12, 137.26, 135.85, 133.24, 131.09, 128.43, 128.24, 127.25, 125.22, 123.89, 123.82, 123.71, 111.04, 59.33, 48.60, 32.55, 32.45, 25.57, 24.86, 24.81, 15.50; Anal for C_24_H_25_N_3_O_4_, Calcd: C, 68.72; H, 6.01; N, 10.02; O, 15.26; found: C, 68.71; H, 5.68; N, 10.03; O, 15.28; MS (70 eV): m/z = 419 (M^+^).

#### *N*-Cyclohexyl-2-(4-methyl-1-oxoisoquinolin-2(1*H*)-yl)-2-(*m*-tolyl)acetamide (7g)

White solid; mp 218–220 °C; ^1^H NMR (400 MHz, DMSO-*d*_6_) δ 8.53 (d, *J* = 7.7 Hz, 1H), 8.32 (dd, *J* = 8.1, 1.4 Hz, 1H), 7.79 (ddd, *J* = 8.3, 7.1, 1.4 Hz, 1H), 7.65 (d, *J* = 8.1 Hz, 1H), 7.57 (ddd, *J* = 8.2, 7.1, 1.2 Hz, 1H), 7.32 (t, *J* = 7.9 Hz, 1H), 7.20 (d, *J* = 7.6 Hz, 1H), 7.09 (d, *J* = 6.1 Hz, 2H), 6.89 (d, *J* = 1.3 Hz, 1H), 6.79 (s, 1H), 3.63 (d, *J* = 7.5 Hz, 1H), 2.30 (s, 3H), 2.12 (d, *J* = 1.2 Hz, 3H), 1.86–1.54 (m, 5H), 1.29–1.03 (m, 5H). ^13^C NMR (101 MHz, DMSO-*d*_6_) δ 167.27, 161.33, 138.73, 137.19, 137.06, 133.09, 129.76, 129.48, 129.37, 128.85, 128.21, 127.06, 126.10, 125.19, 123.79, 110.10, 59.71, 48.48, 32.62, 32.53, 25.61, 24.94, 24.87, 21.48, 15.69; Anal for C_25_H_28_N_2_O_2_, Calcd: C, 77.29; H, 7.26; N, 7.21; O, 8.24; found: C, 77.33; H, 7.28; N, 7.18; O, 8.21; MS (70 eV): m/z = 388 (M^+^).

#### *N*-(Tert-butyl)-2-(4-methoxyphenyl)-2-(4-methyl-1-oxoisoquinolin-2(1*H*)-yl)acetamide (7h)

White solid; mp 196–198 °C; ^1^H NMR (400 MHz, DMSO-*d*_6_) δ 8.33–8.23 (m, 2H), 7.75 (ddd, *J* = 8.3, 7.0, 1.4 Hz, 1H), 7.61 (d, *J* = 8.1 Hz, 1H), 7.53 (ddd, *J* = 8.1, 7.0, 1.1 Hz, 1H), 7.24–7.17 (m, 2H), 7.01–6.93 (m, 2H), 6.81 (d, *J* = 1.3 Hz, 1H), 6.74 (s, 1H), 3.75 (s, 3H), 2.09 (d, *J* = 1.2 Hz, 3H), 1.26 (s, 9H). ^13^C NMR (101 MHz, DMSO-*d*_6_) δ 167.98, 161.31, 159.54, 137.19, 133.00, 130.56, 129.05, 128.95, 128.19, 126.95, 125.23, 123.73, 114.84, 109.81, 59.50, 55.61, 51.11, 28.83, 15.71; Anal for C_23_H_26_N_2_O_3_, Calcd: C, 72.99; H, 6.92; N, 7.40; O, 12.68; found: C, 73.03; H, 6.90; N, 7.36; O, 12.70; MS (70 eV): m/z = 378 (M^+^).

#### *N*-(Tert-butyl)-2-(4-methyl-1-oxoisoquinolin-2(1*H*)-yl)-2-(*p*-tolyl)acetamide (7i)

White solid; mp 200–202 °C; ^1^H NMR (400 MHz, DMSO-*d*_6_) δ 8.34–8.25 (m, 2H), 7.75 (ddd, *J* = 8.3, 7.0, 1.4 Hz, 1H), 7.60 (d, *J* = 8.0 Hz, 1H), 7.53 (ddd, *J* = 8.1, 7.1, 1.1 Hz, 1H), 7.25–7.10 (m, 4H), 6.82 (d, *J* = 1.3 Hz, 1H), 6.78 (s, 1H), 2.29 (s, 3H), 2.08 (d, *J* = 1.2 Hz, 3H), 1.26 (s, 9H). ^13^C NMR (101 MHz, DMSO-*d*_6_) δ 167.83, 161.33, 138.10, 137.19, 134.39, 133.02, 130.03, 129.09, 129.02, 128.21, 126.96, 125.21, 123.73, 109.85, 59.61, 51.13, 28.82, 21.18, 15.69; Anal for C_23_H_26_N_2_O_2_, Calcd: C, 76.21; H, 7.23; N, 7.73; O, 8.83; found: C, 76.18; H, 7.25; N, 7.69; O, 8.87; MS (70 eV): m/z = 362 (M^+^).

#### *N*-(Tert-butyl)-2-(4-methyl-1-oxoisoquinolin-2(1*H*)-yl)-2-(3-nitrophenyl)acetamide (7j)

Yellow solid; ^1^H NMR (400 MHz, DMSO-*d*_6_) δ 8.50 (s, 1H), 8.33 (dd, *J* = 8.0, 1.4 Hz, 1H), 8.26 (dt, *J* = 7.1, 2.3 Hz, 1H), 8.14 (d, *J* = 2.5 Hz, 1H), 7.81 (ddd, *J* = 8.4, 7.1, 1.4 Hz, 1H), 7.76–7.69 (m, 2H), 7.66 (d, *J* = 8.0 Hz, 1H), 7.58 (td, *J* = 7.5, 1.1 Hz, 1H), 6.96 (s, 1H), 6.95 (d, *J* = 1.3 Hz, 1H), 2.14 (d, *J* = 1.2 Hz, 3H), 1.32 (s, 9H). ^13^C NMR (101 MHz, DMSO-*d*_6_) δ 166.88, 161.38, 148.45, 139.43, 137.26, 135.90, 133.24, 131.13, 128.65, 128.26, 127.20, 125.16, 123.85, 123.79, 123.68, 110.75, 59.21, 51.41, 28.72, 15.49; Anal for C_22_H_23_N_3_O_4_, Calcd: C, 67.16; H, 5.89; N, 10.68; O, 16.27; found: C, 67.20; H, 5.91; N, 10.65; O, 16.24; MS (70 eV): m/z = 393 (M^+^).

## Conclusion

In this paper, a novel catalyst is synthesized and characterized based on the immobilization of palladium onto Fucus Vesiculosus extract. For better performance of the catalyst and more facile separation from the reaction mixture, Pd@m*FuVe* catalyst was modified by magnetic nanoparticles. The catalyst was characterized by several characterization techniques and all the results confirmed the successful synthesis of Pd@m*FuVe* catalyst. The catalyst was used for the synthesis of *N*-alkyl-2-(4-methyl-1-oxoisoquinolin-2(1*H*)-yl)-2-phenylacetamide derivatives. The products were synthesized in three steps. In the first step, 2-iodobenzoic acid was synthesized from 2-aminobenzoic acid. which participated in a multicomponent reaction with allylamine, aldehydes, and isocyanides. Finally, the cyclization reaction of compound **6** in the presence of Pd@m*FuVe* catalyst leads to the synthesis of *N*-alkyl-2-(4-methyl-1-oxoisoquinolin-2(1*H*)-yl)-2-phenylacetamides. As an advantage, the products are obtained in high isolated yields. In addition, the catalyst showed very good reusability after 5 sequential runs. The most significant, Pd@m*FuVe* catalyst is fabricated from a plant extract source as a green support for the catalyst.

## Supplementary Information

Supplementary Figures.

## Data Availability

All data generated or analyzed during this study are included in this published article. The copies of ^1^H NMR and ^13^C NMR spectrum of the compounds are available as Supplementary Information.
