# Supplementary material for: Palladium supported magnetic Fucus Vesiculosus extract as a natural and novel catalyst for the synthesis of N-alkyl-2-(4-methyl-1-oxoisoquinolin-2(1H)-yl)-2-phenylacetamide derivatives
Source: Sci Rep. 2023 Jan 23;13:1272. doi: 10.1038/s41598-023-28121-1 (PMC9870856; doi:10.1038/s41598-023-28121-1)
Supplement: Supplementary file 1 — Supplementary Figures. [file 41598_2023_28121_MOESM1_ESM.pdf]

Palladium Supported Magnetic Fucus Vesiculosus Extract as a Natural and Novel Catalyst for the Synthesis of *N*-Alkyl-2-(4-methyl-1-oxoisoquinolin-2(1*H*)-yl)-2-phenylacetamide Derivatives

Faeze Yousefnejad[1], Saeed Bahadorikhalili[2], Maryam Esmkhani[1], Mehdi Adib[1,*], Shahrzad Javanshir[3], Samanehsadat Hosseini[4], Bagher Larijani[5], Mohammad Mahdavi[5,*]

[1] School of Chemistry, College of Sciences, University of Tehran, Tehran, Iran, email: madib@ut.ac.ir.

[2] Department of Electronic Engineering, Universitat Rovira i Virgili, 43007, Tarragona, Spain

[3] Department of Chemistry, Iran University of Science and Technology, Tehran, Iran.

[4] Shahid Beheshti University of Medical Sciences, Tehran, Iran.

[5] Endocrinology and Metabolism Research Center, Endocrinology and Metabolism Clinical Sciences Institute, Tehran University of Medical Sciences, Tehran, Iran, email: momahdavi@sina.tums.ac.ir.

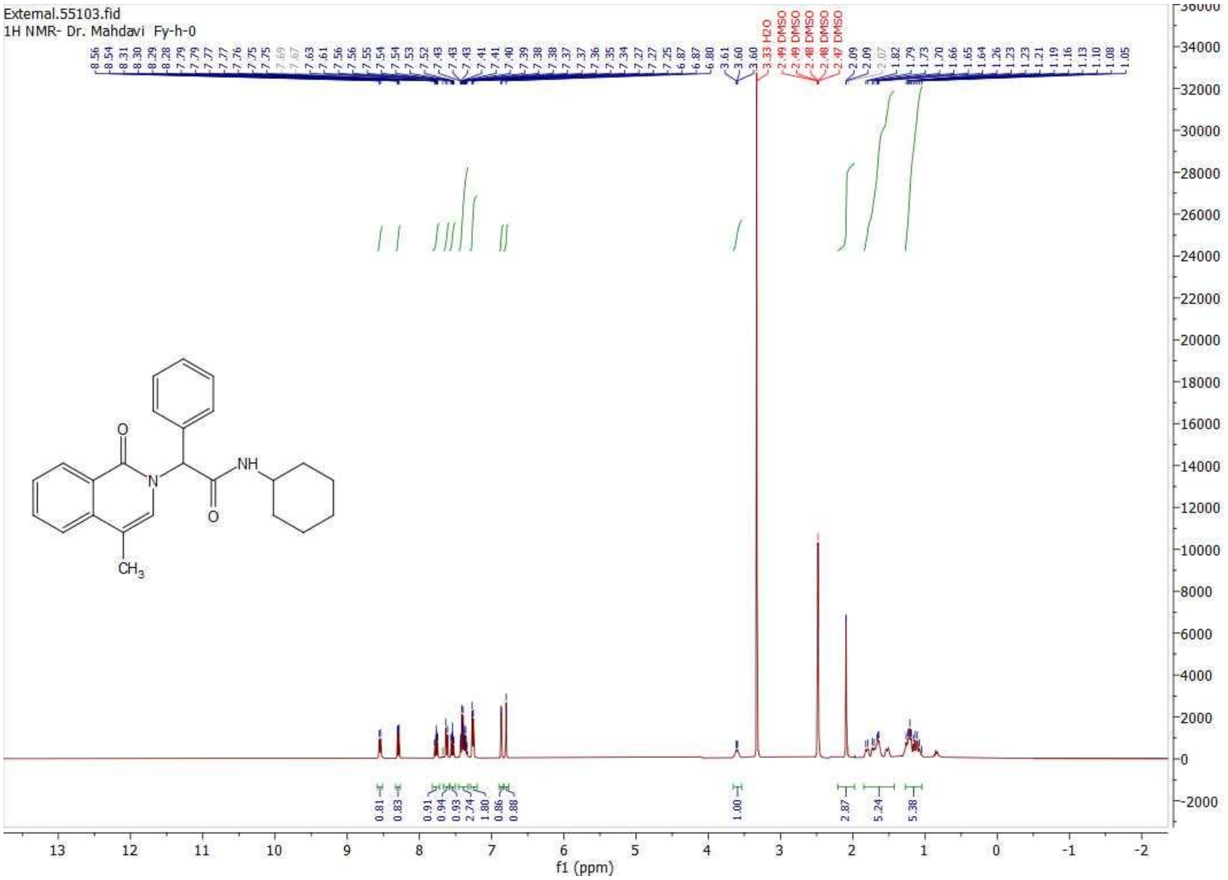

Figure S1. ^{1}H NMR spectra of *N*-cyclohexyl-2-(4-methyl-1-oxoisoquinolin-2(1*H*)-yl)-2-phenylacetamide (**7a**)

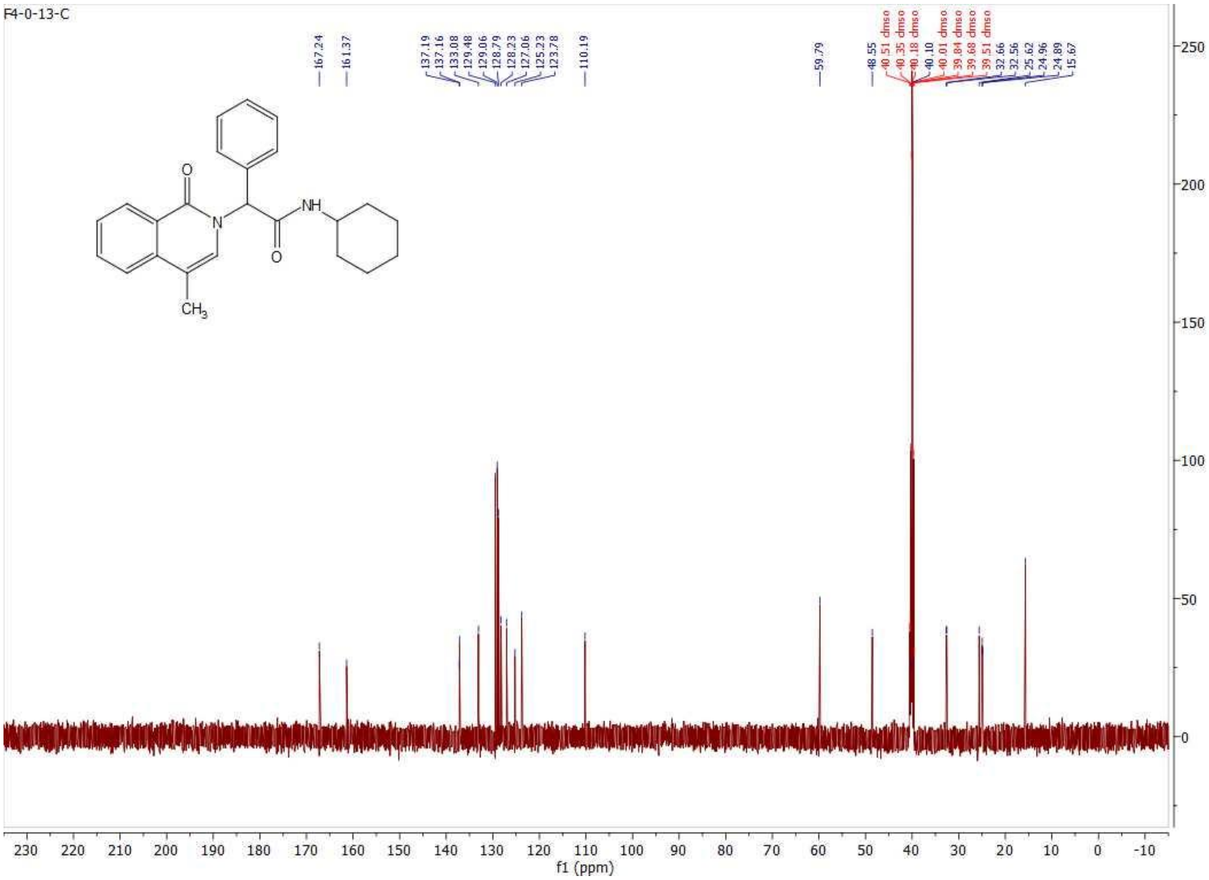

Figure S2. ^{13}C NMR spectra of *N*-cyclohexyl-2-(4-methyl-1-oxoisoquinolin-2(1*H*)-yl)-2-phenylacetamide (**7a**)

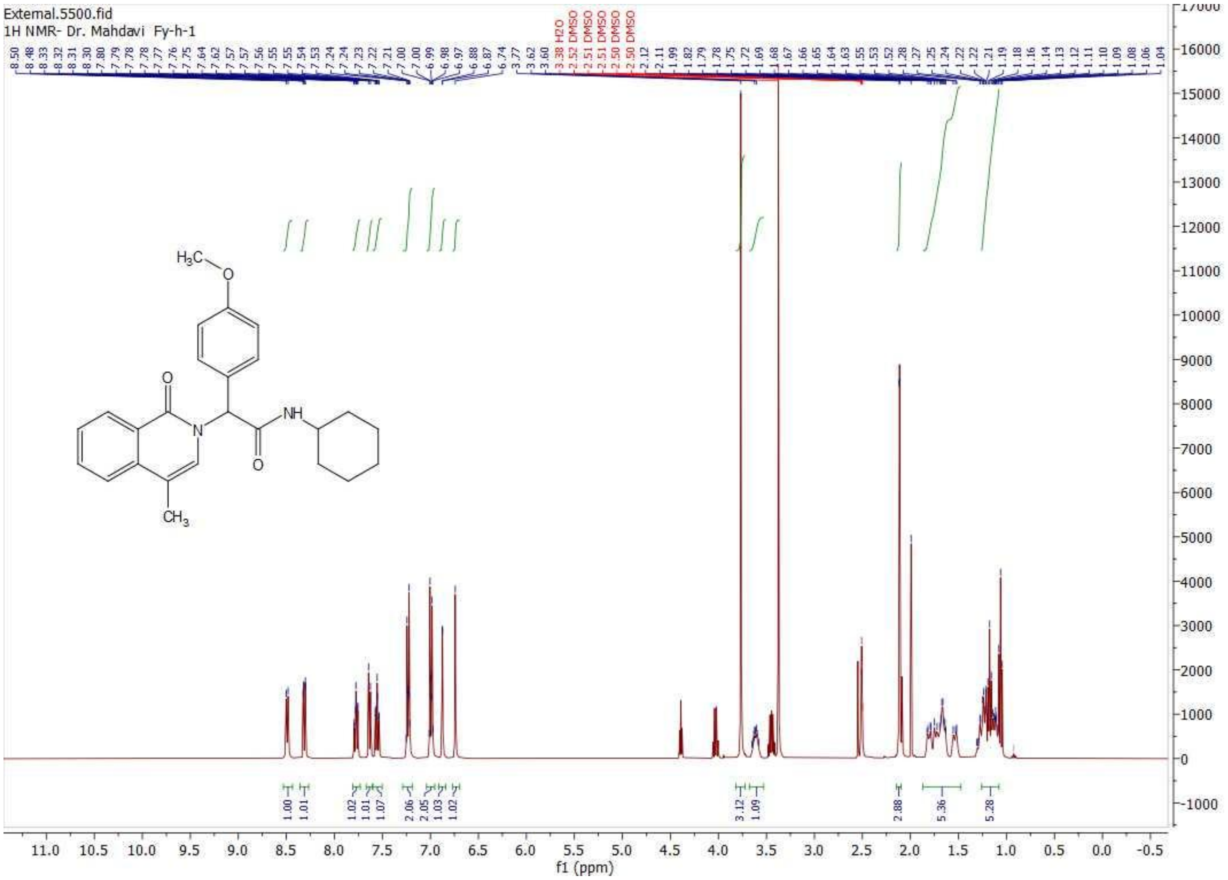

Figure S3. ^{1}H NMR spectra of *N*-cyclohexyl-2-(4-methoxyphenyl)-2-(4-methyl-1-oxoisoquinolin-2(1*H*)-yl)acetamide (**7b**)

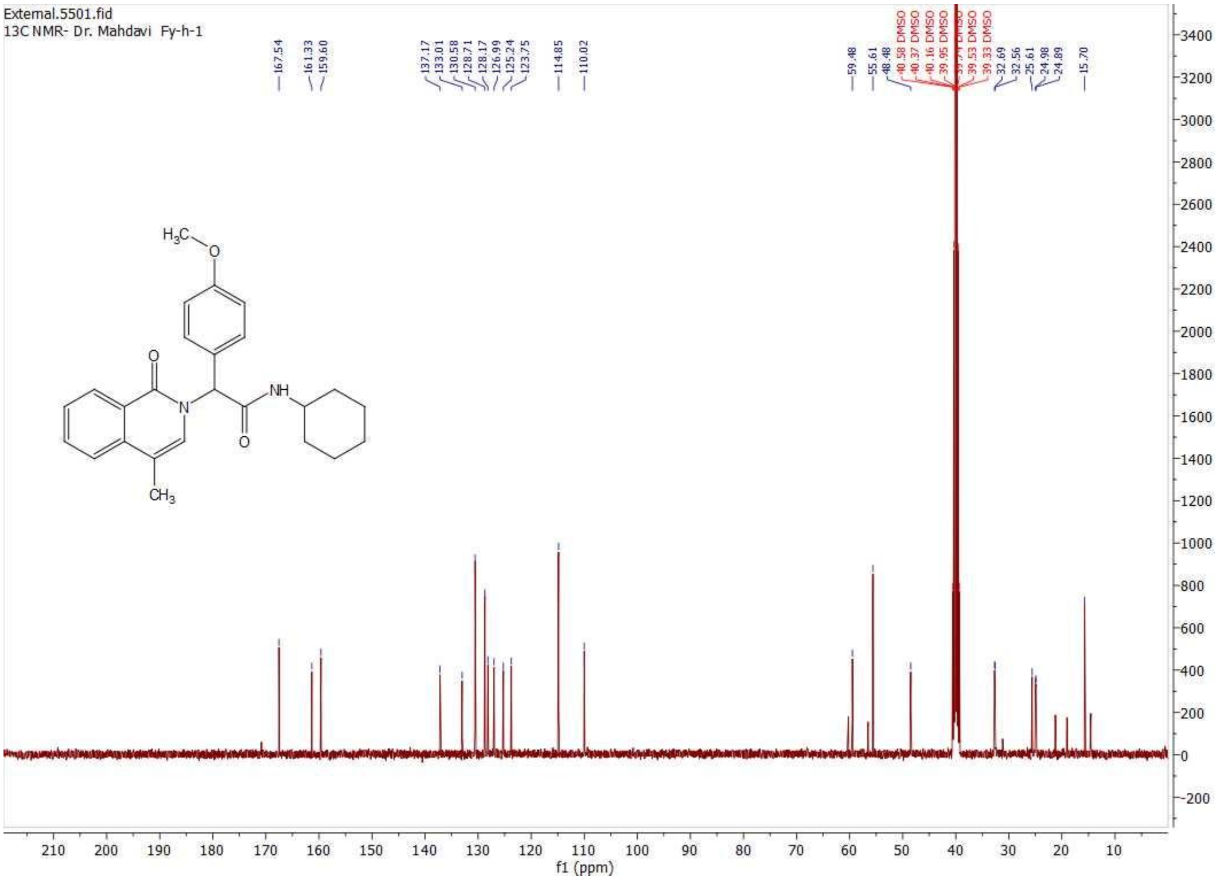

Figure S4. ^{13}C NMR spectra of *N*-cyclohexyl-2-(4-methoxyphenyl)-2-(4-methyl-1-oxoisoquinolin-2(1*H*)-yl)acetamide (**7b**)

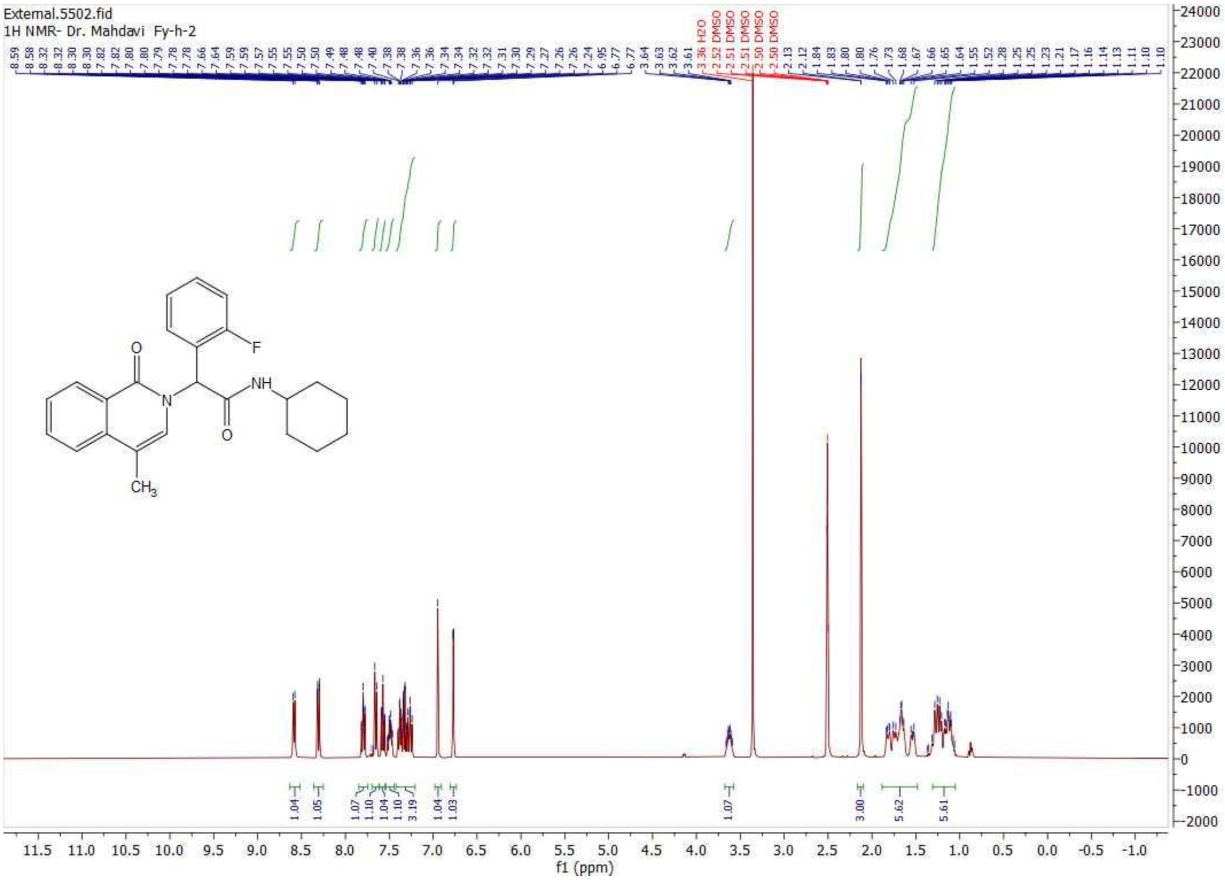

Figure S5. ^{1}H NMR spectra of *N*-cyclohexyl-2-(2-fluorophenyl)-2-(4-methyl-1-oxoisoquinolin-2(1*H*)-yl)acetamide (**7c**)

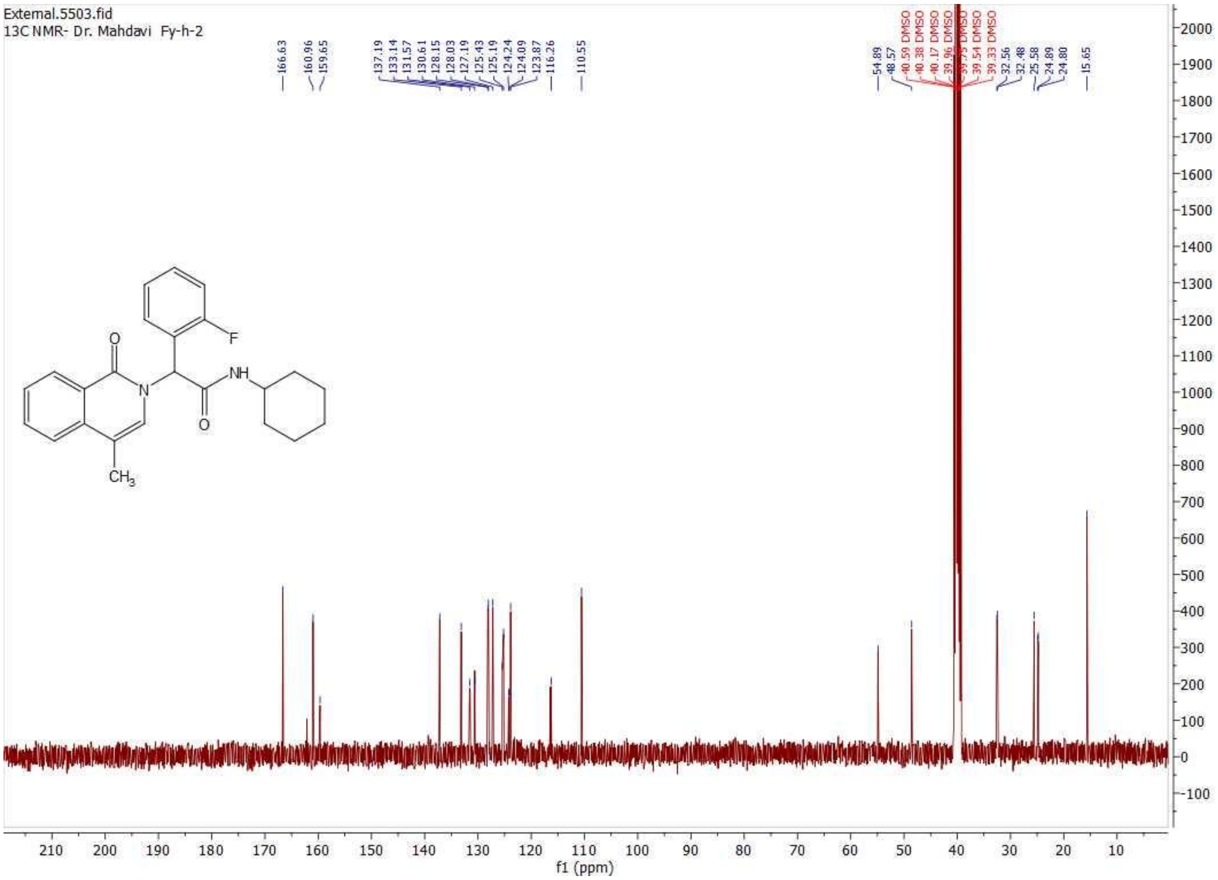

Figure S6. ^{13}C NMR spectra of *N*-cyclohexyl-2-(2-fluorophenyl)-2-(4-methyl-1-oxoisoquinolin-2(1*H*)-yl)acetamide (**7c**)

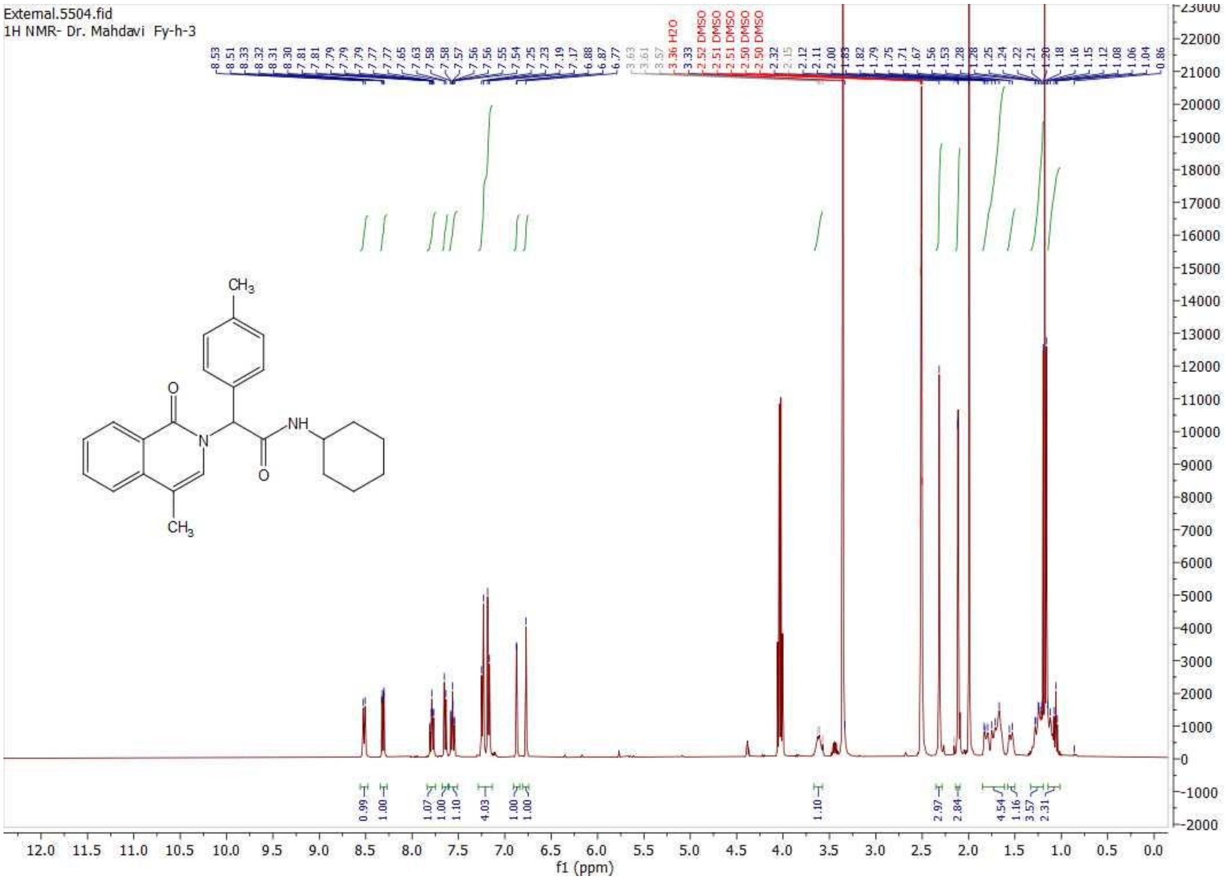

Figure S7. ^{1}H NMR spectra of *N*-cyclohexyl-2-(4-methyl-1-oxoisoquinolin-2(1*H*)-yl)-2-(p-tolyl)acetamide (**7d**)

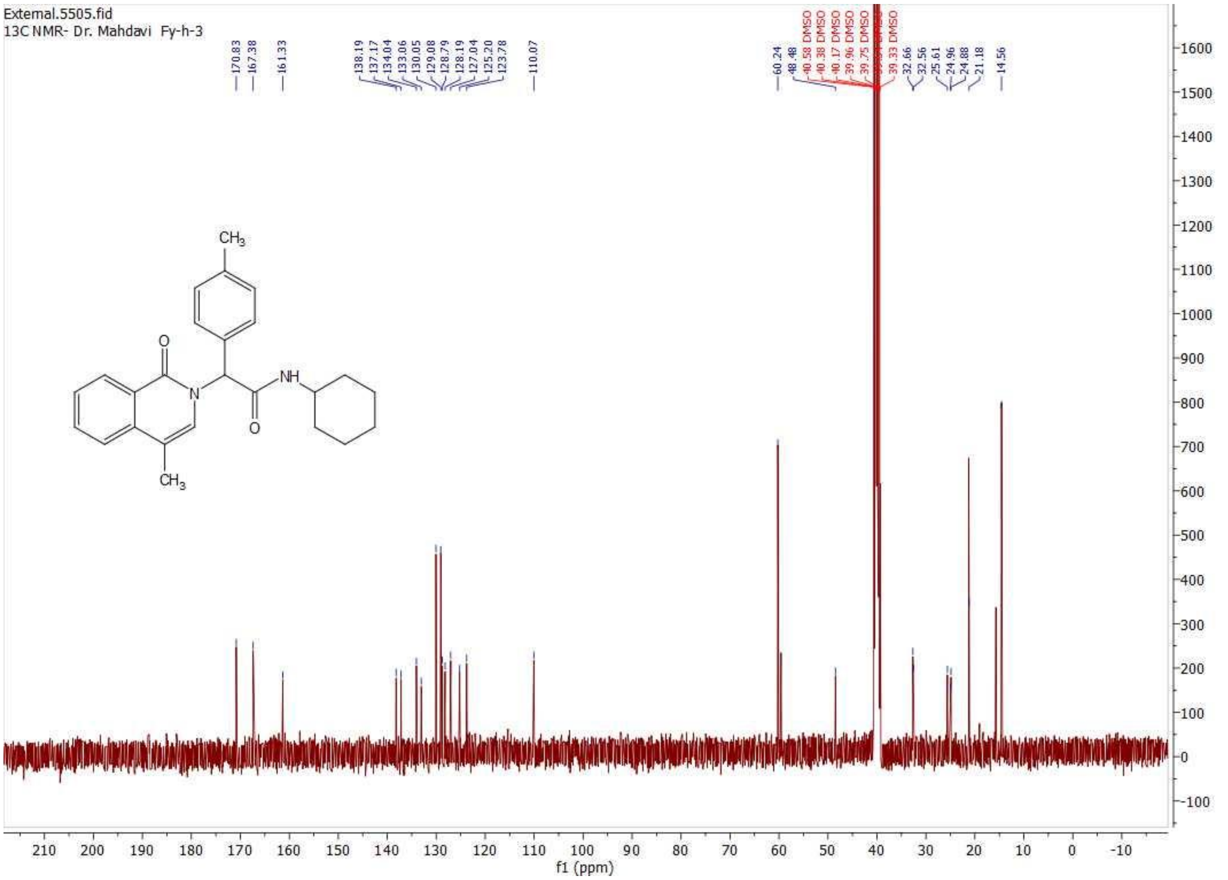

Figure S8. ^{13}C NMR spectra of *N*-cyclohexyl-2-(4-methyl-1-oxoisoquinolin-2(1*H*)-yl)-2-(p-tolyl)acetamide (**7d**)

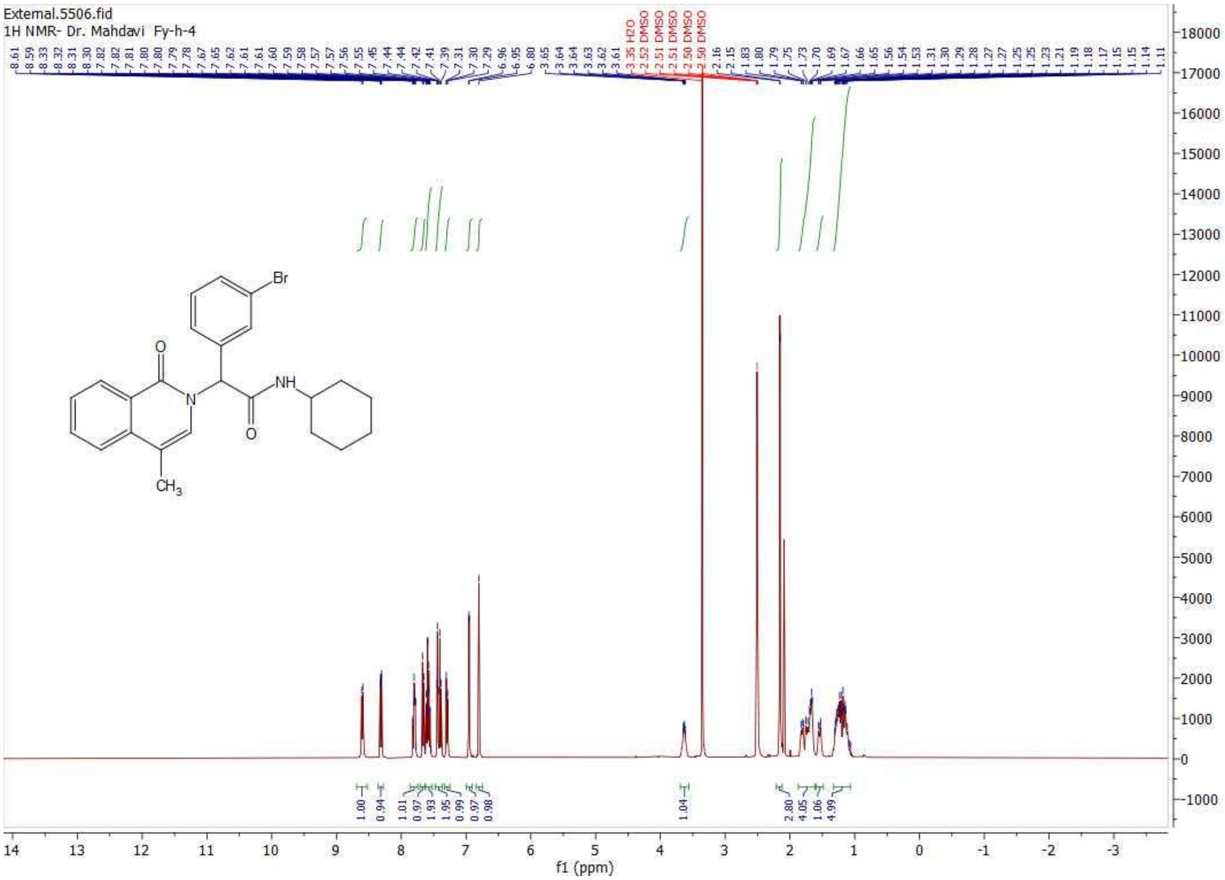

Figure S9. ^{1}H NMR spectra of 2-(3-bromophenyl)-*N*-cyclohexyl-2-(4-methyl-1-oxoisoquinolin-2(1*H*)-yl)acetamide (**7e**)

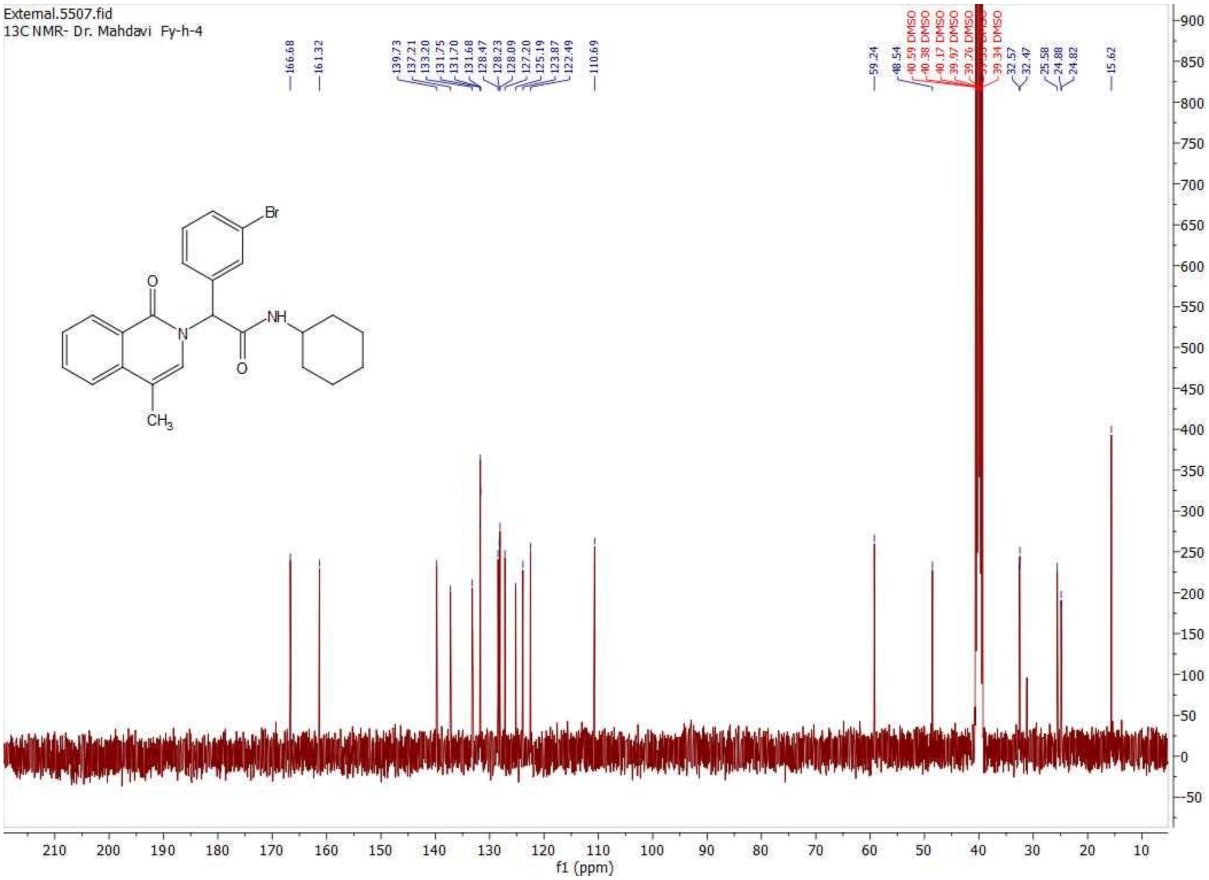

Figure S10. ^{13}C NMR spectra of 2-(3-bromophenyl)-*N*-cyclohexyl-2-(4-methyl-1-oxoisoquinolin-2(1*H*)-yl)acetamide (**7e**)

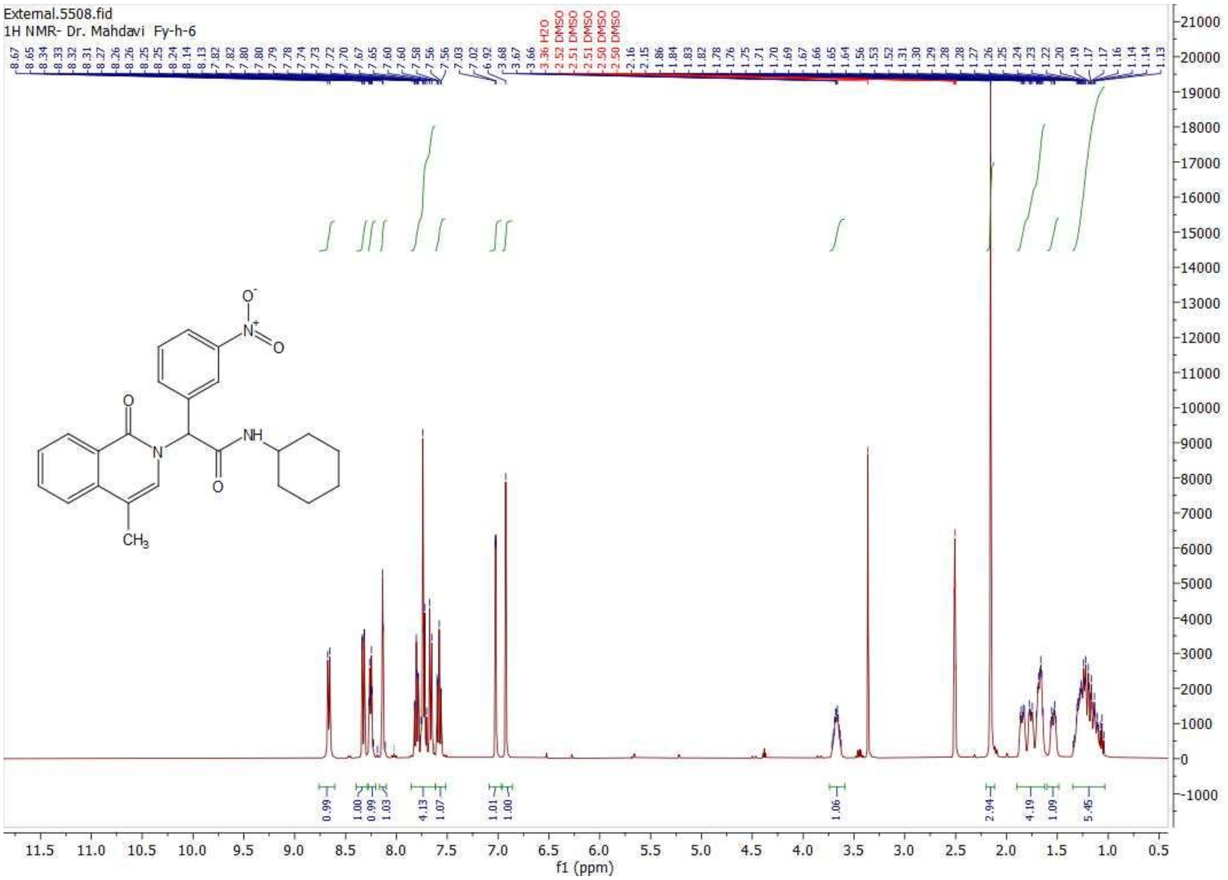

Figure S11. ^{1}H NMR spectra of *N*-cyclohexyl-2-(4-methyl-1-oxoisoquinolin-2(1*H*)-yl)-2-(3-nitrophenyl)acetamide (**7f**)

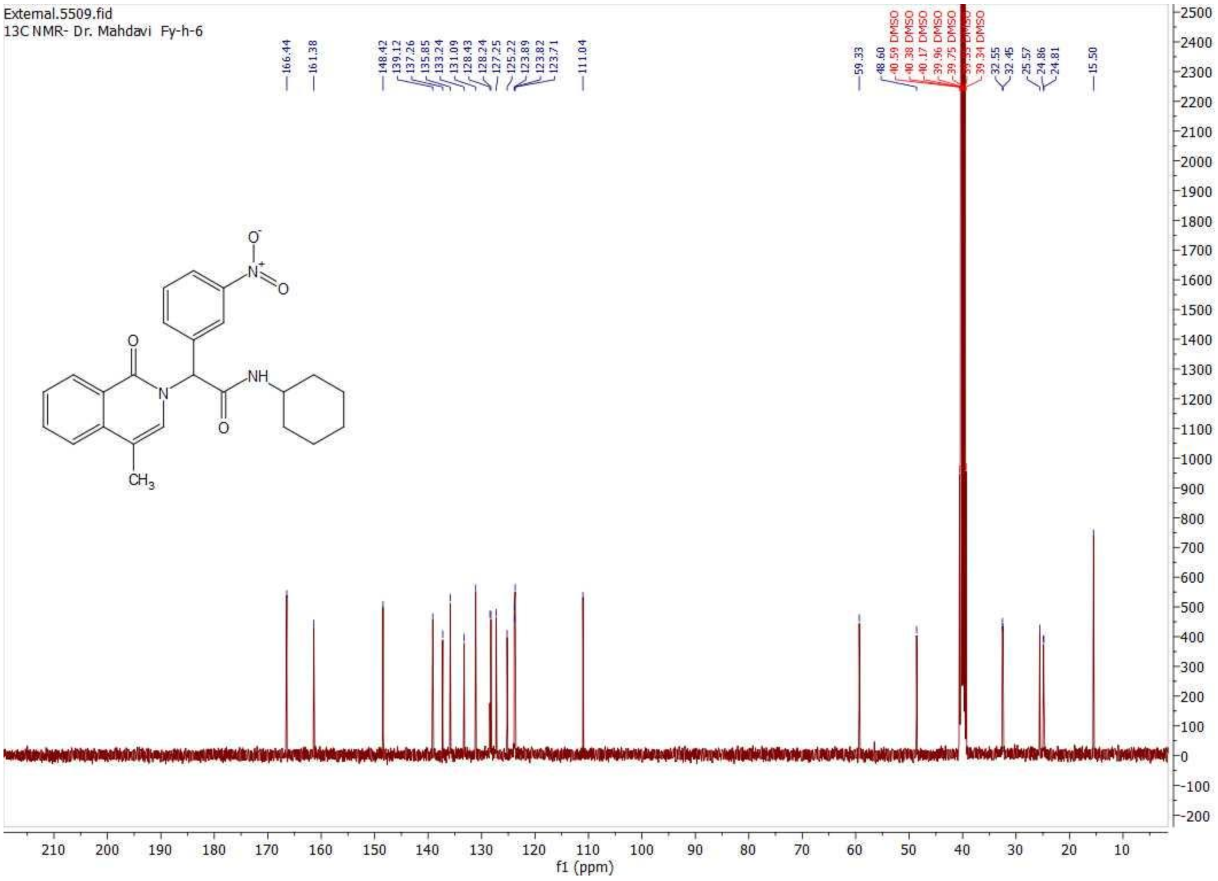

Figure S12. ^{13}C NMR spectra of *N*-cyclohexyl-2-(4-methyl-1-oxoisoquinolin-2(1*H*)-yl)-2-(3-nitrophenyl)acetamide (**7f**)

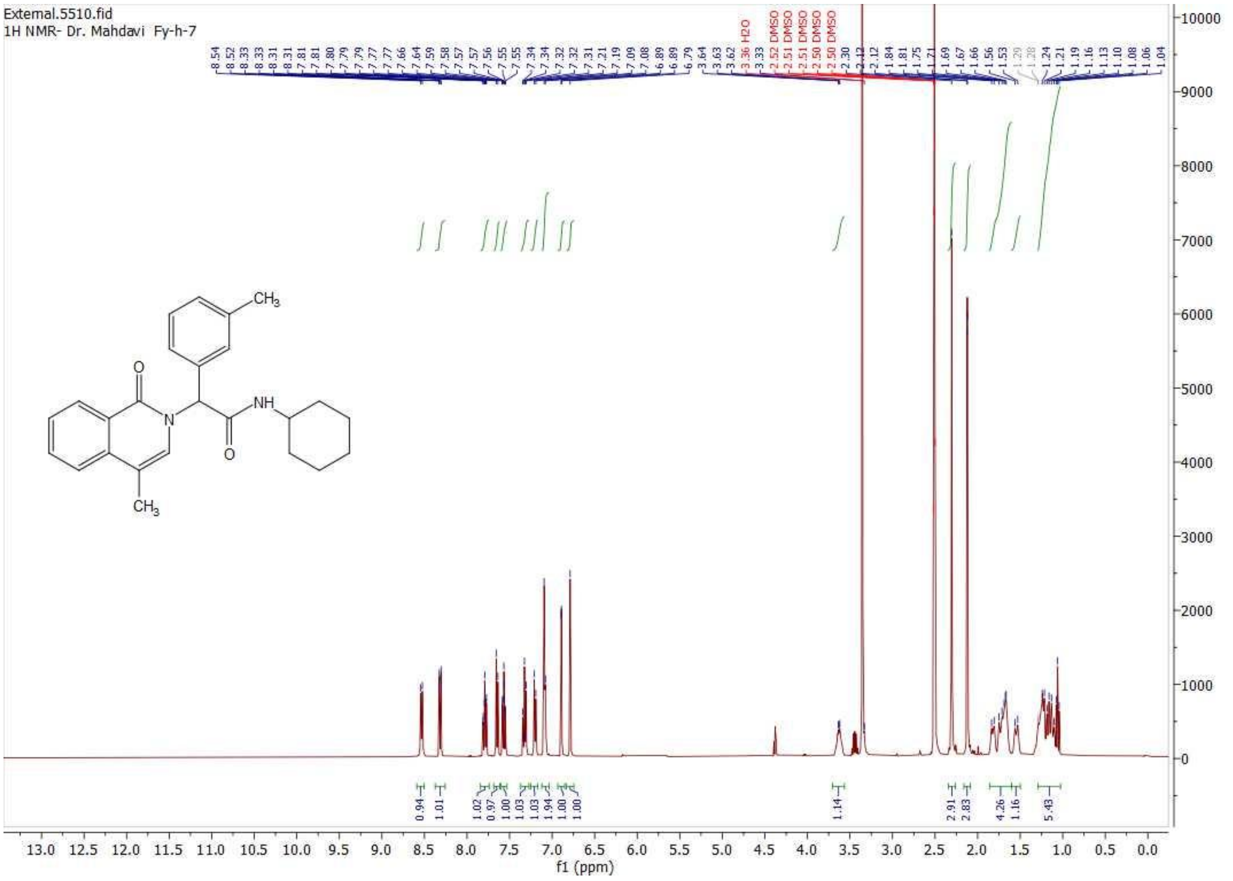

Figure S13. ^{1}H NMR spectra of *N*-cyclohexyl-2-(4-methyl-1-oxoisoquinolin-2(1*H*)-yl)-2-(m-tolyl)acetamide (**7g**)

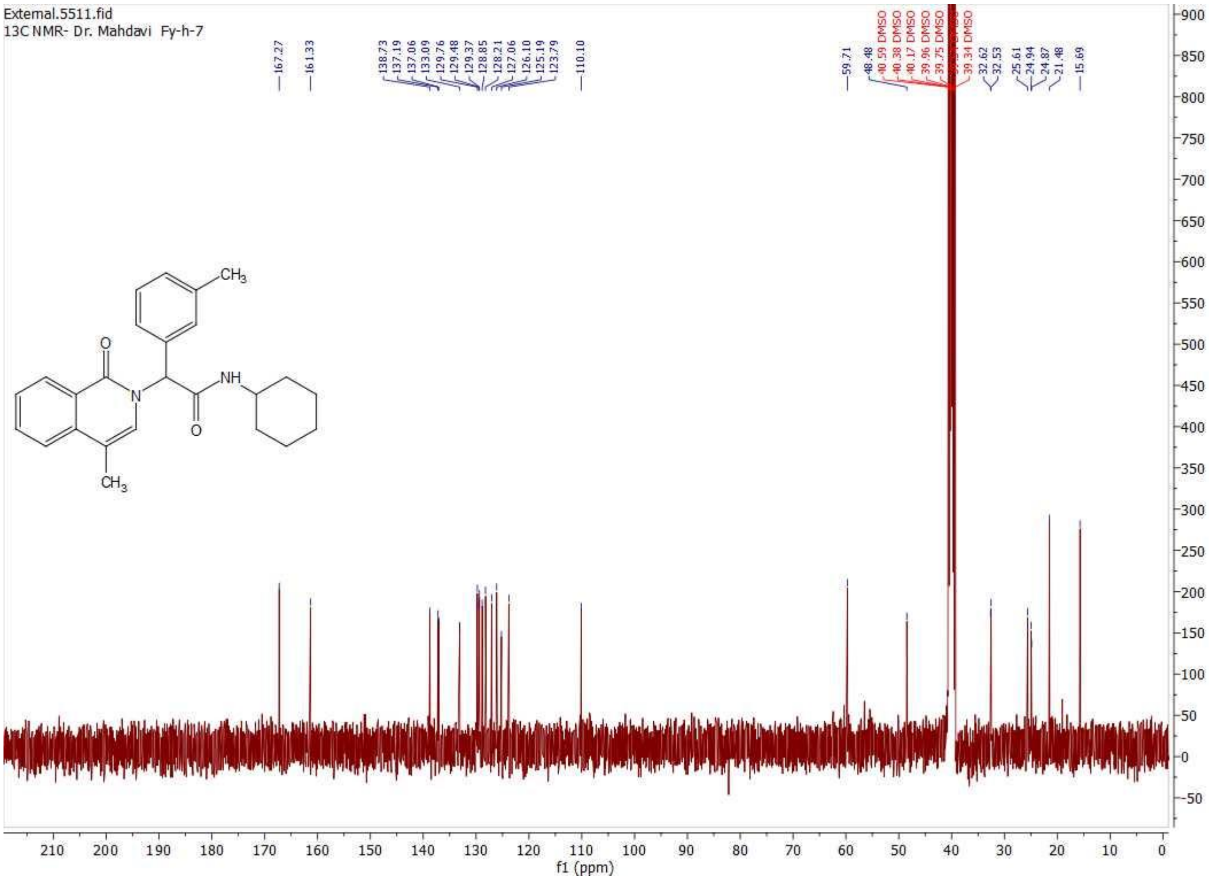

Figure S14. ^{13}C NMR spectra of *N*-cyclohexyl-2-(4-methyl-1-oxoisoquinolin-2(1*H*)-yl)-2-(m-tolyl)acetamide (**7g**)

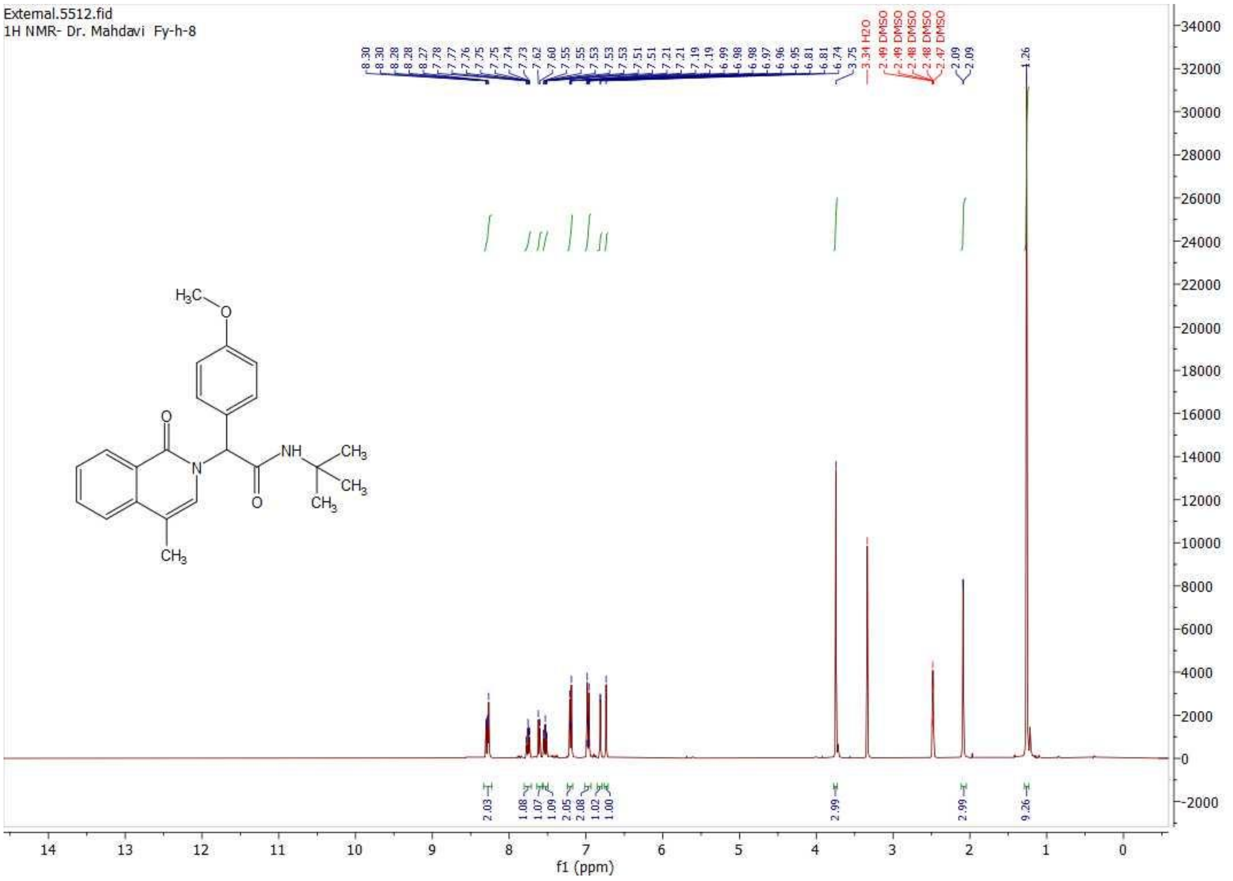

Figure S15. ^{1}H NMR spectra of *N*-(tert-butyl)-2-(4-methoxyphenyl)-2-(4-methyl-1-oxoisoquinolin-2(1*H*)-yl)acetamide (**7h**)

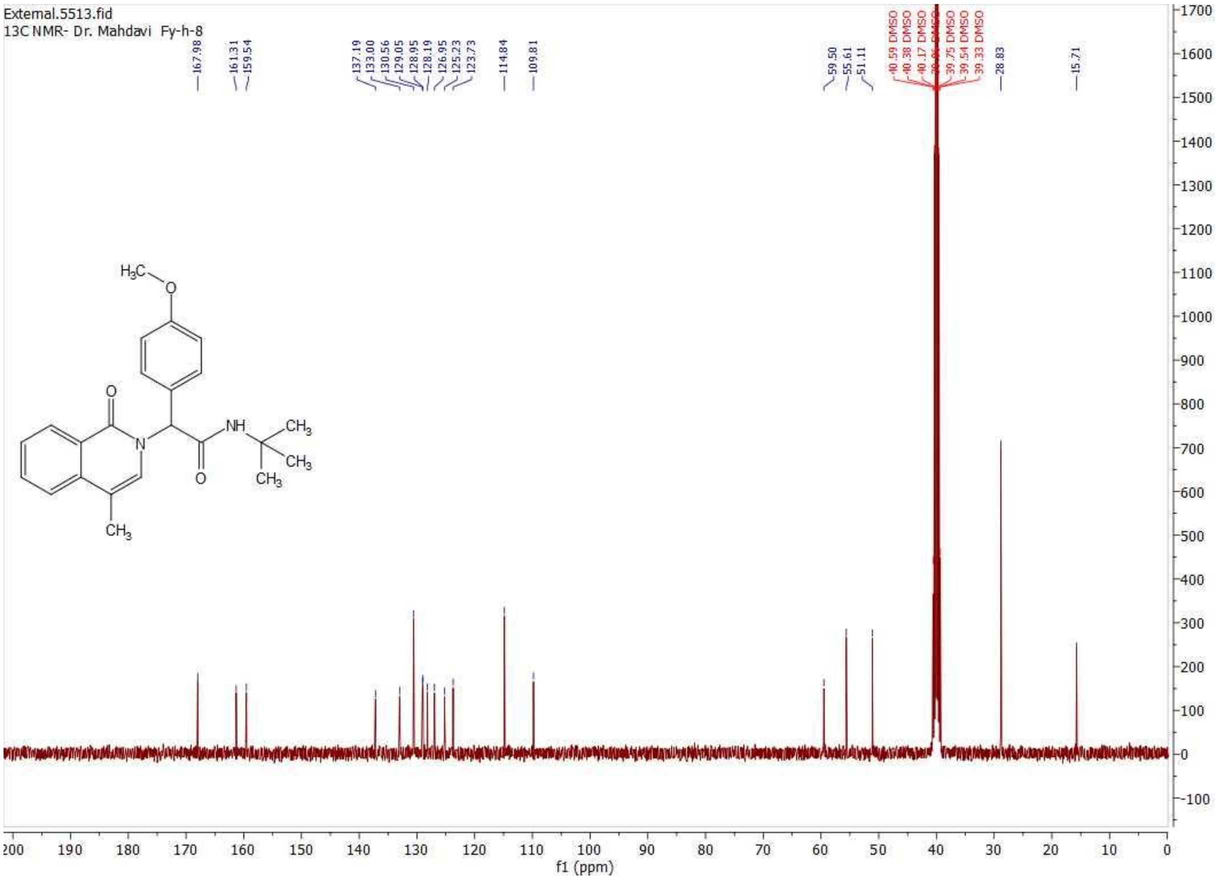

Figure S16. ^{13}C NMR spectra of *N*-(tert-butyl)-2-(4-methoxyphenyl)-2-(4-methyl-1-oxoisoquinolin-2(1*H*)-yl)acetamide (**7h**)

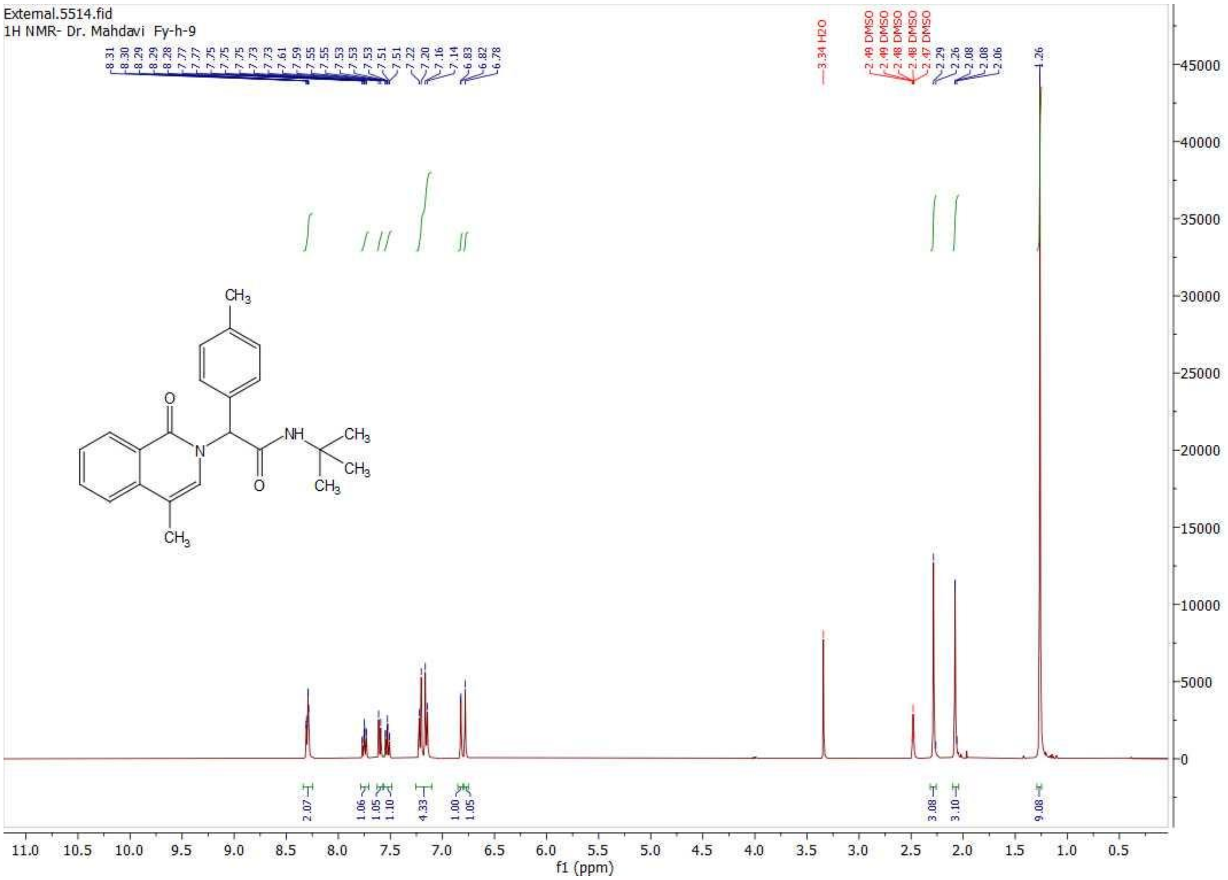

Figure S17. ^{1}H NMR spectra of *N*-(tert-butyl)-2-(4-methyl-1-oxoisoquinolin-2(1*H*)-yl)-2-(p-tolyl)acetamide (**7i**)

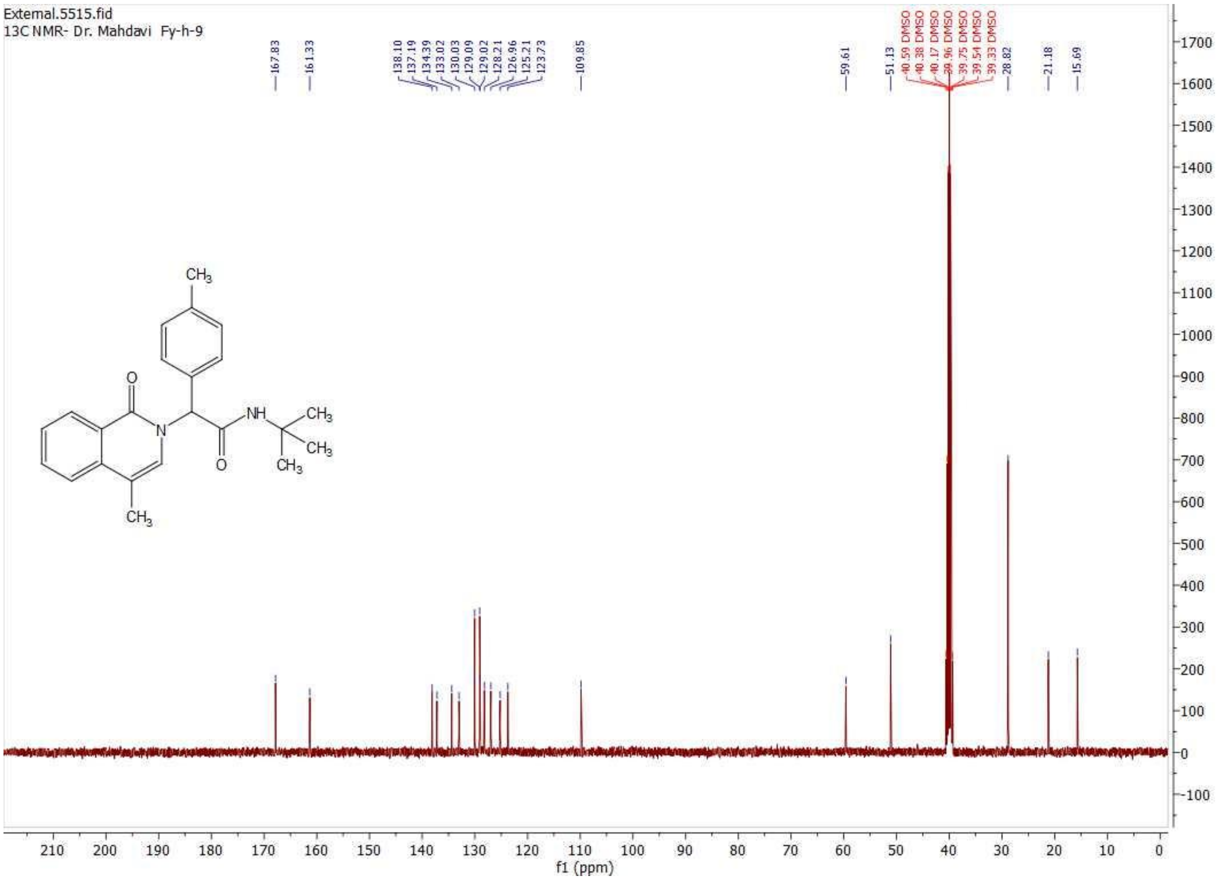

Figure S18. ^{13}C NMR spectra of *N*-(tert-butyl)-2-(4-methyl-1-oxoisoquinolin-2(1*H*)-yl)-2-(p-tolyl)acetamide (**7i**)

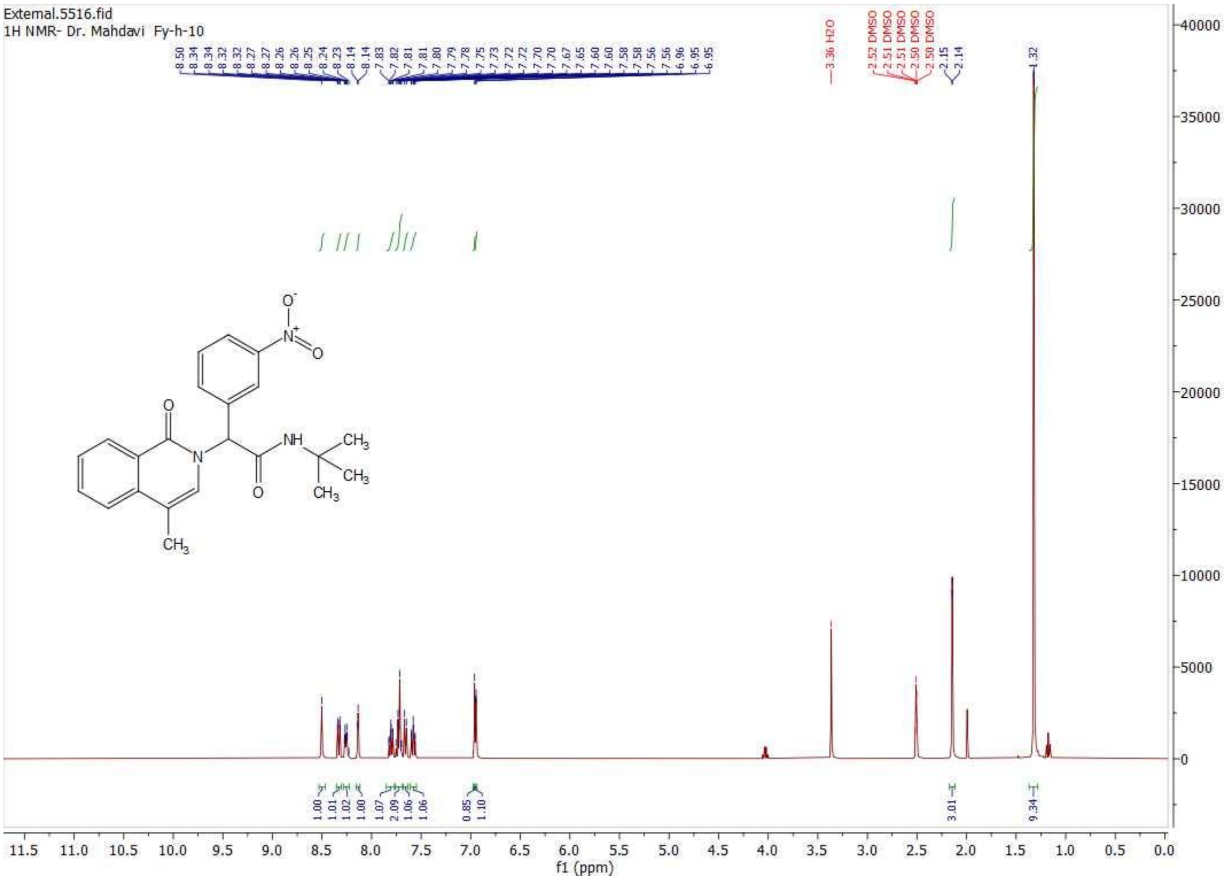

Figure S19. ^{1}H NMR spectra of *N*-(tert-butyl)-2-(4-methyl-1-oxoisoquinolin-2(1*H*)-yl)-2-(3-nitrophenyl)acetamide (**7j**)

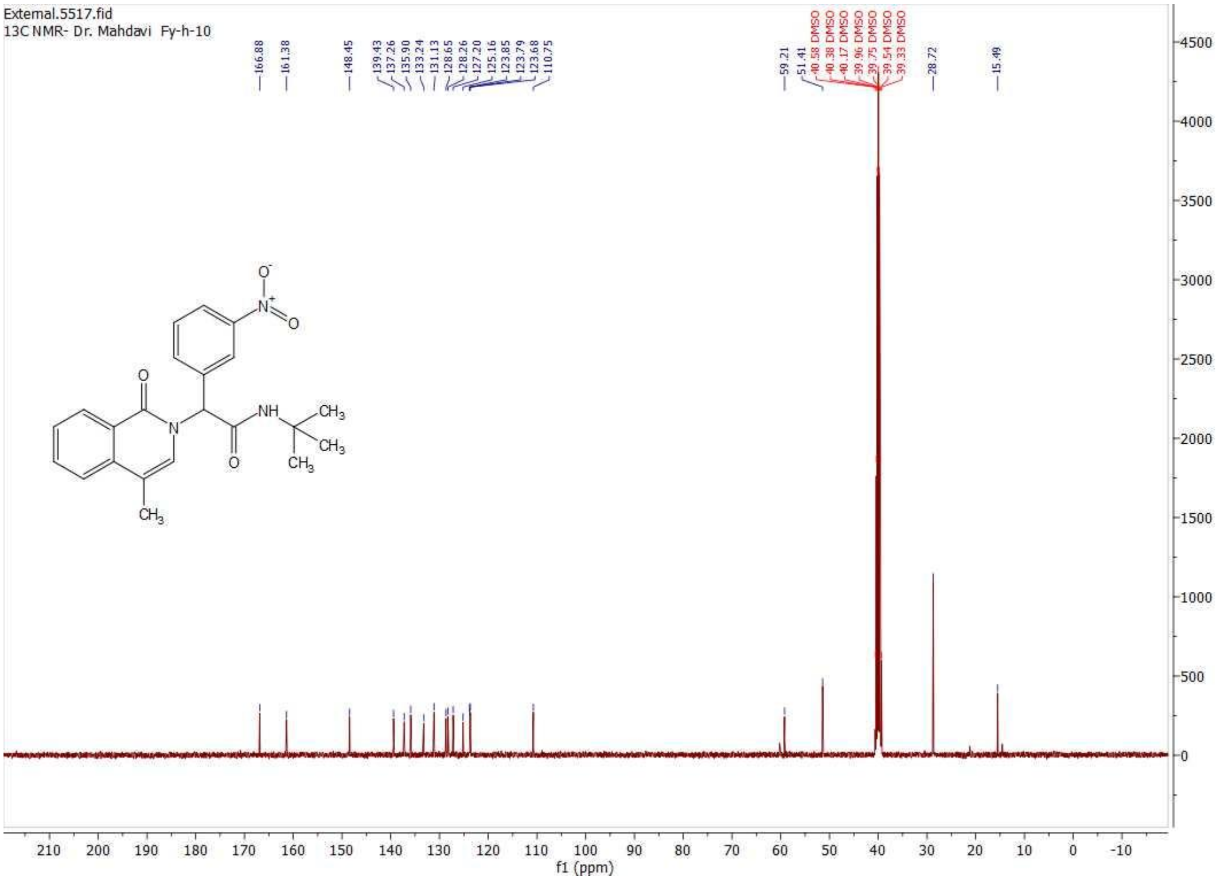

Figure S20. ^{13}C NMR spectra of *N*-(tert-butyl)-2-(4-methyl-1-oxoisoquinolin-2(1*H*)-yl)-2-(3-nitrophenyl)acetamide (**7j**)
